# The *Lr34* adult plant rust resistance gene provides seedling resistance in durum wheat without senescence

**DOI:** 10.1111/pbi.12684

**Published:** 2017-03-10

**Authors:** Amy Rinaldo, Brian Gilbert, Rainer Boni, Simon G. Krattinger, Davinder Singh, Robert F. Park, Evans Lagudah, Michael Ayliffe

**Affiliations:** ^1^ CSIRO AgricultureCanberraACTAustralia; ^2^ Department of Plant and Microbial BiologyUniversity of ZurichZurichSwitzerland; ^3^ Plant Breeding InstituteUniversity of SydneyNarellanNSWAustralia

**Keywords:** *Puccinia*, *Triticum*, rust, ABC transporter, *Blumeria*

## Abstract

The hexaploid wheat (*Triticum aestivum*) adult plant resistance gene, *Lr34/Yr18/Sr57/Pm38/Ltn1*, provides broad‐spectrum resistance to wheat leaf rust (*Lr34*), stripe rust (*Yr18*), stem rust (*Sr57*) and powdery mildew (*Pm38*) pathogens, and has remained effective in wheat crops for many decades. The partial resistance provided by this gene is only apparent in adult plants and not effective in field‐grown seedlings. *Lr34* also causes leaf tip necrosis (*Ltn1*) in mature adult plant leaves when grown under field conditions. This D genome‐encoded bread wheat gene was transferred to tetraploid durum wheat (*T. turgidum*) cultivar Stewart by transformation. Transgenic durum lines were produced with elevated gene expression levels when compared with the endogenous hexaploid gene. Unlike nontransgenic hexaploid and durum control lines, these transgenic plants showed robust seedling resistance to pathogens causing wheat leaf rust, stripe rust and powdery mildew disease. The effectiveness of seedling resistance against each pathogen correlated with the level of transgene expression. No evidence of accelerated leaf necrosis or up‐regulation of senescence gene markers was apparent in these seedlings, suggesting senescence is not required for *Lr34* resistance, although leaf tip necrosis occurred in mature plant flag leaves. Several abiotic stress‐response genes were up‐regulated in these seedlings in the absence of rust infection as previously observed in adult plant flag leaves of hexaploid wheat. Increasing day length significantly increased *Lr34* seedling resistance. These data demonstrate that expression of a highly durable, broad‐spectrum adult plant resistance gene can be modified to provide seedling resistance in durum wheat.

## Introduction

Wheat rust diseases caused by *Puccinia graminis* f. sp. *tritici* (stem rust), *P. striiformis* f. sp. *tritici* (stripe/yellow rust) and *P. triticina* (leaf rust) remain a major threat to world production of hexaploid bread wheat (*Triticum aestivum* L., 2*n* = 6*x* = AABBDD) and tetraploid durum (pasta) wheat (*T. turgidum* L. subsp. *durum*, 2*n* = 4*x* = 28, AABB) (Chen *et al*., 2014; Huerta‐Espino *et al*., 2011; Kolmer, 2005; Singh *et al*., 2011a). Resistance to these wheat rust pathogens is achieved most economically using resistance genes derived from wheat landraces and wild relative species. However, most wheat rust resistance genes are ultimately overcome by pathogen evolution to virulence.

Wheat rust resistance genes have been broadly categorized into two groups, all‐stage or seedling resistance genes and adult plant resistance (APR) genes. All‐stage resistance genes, function at all stages of plant development and although often race specific, can provide high levels of resistance. Nine cloned all‐stage wheat rust resistance genes each encode a nucleotide‐binding site leucine‐rich repeat protein (NLR), a large class of disease resistance proteins characterized in numerous plant species (Cloutier *et al*., 2007; Feuillet *et al*., 2003; Huang *et al*., 2003; Liu *et al*., 2014; Mago *et al*., 2015; Periyannan *et al*., 2013; Saintenac *et al*., 2013; Steuernagel *et al*., 2016). These proteins recognize pathogen molecules (effectors) introduced into plant cells or alternatively effector‐mediated modifications of host proteins, leading to defence activation (reviewed by Dodds and Rathjen, 2010). Pathogen effector genes can rapidly evolve to avoid plant recognition, making NLR resistance genes ineffective.

Unlike all‐stage resistance, APR occurs in mature wheat plants only and tends to provide partial resistance, although specific APR gene combinations show additive resistance effects (Singh *et al*., 2011b). Some APR genes provide resistance to all isolates of a pathogen species (broad spectrum) and in some cases resistance to multiple pathogen species. For example, the *Lr34/Yr18/Sr57/Pm38/Ltn1* gene (hereafter called *Lr34*) provides resistance to *P. triticina* (*Lr*), *P. striiformis* f. sp. *tritici* (*Yr*), *P. graminis* f. sp. *tritici* (*Sr*) and *Blumeria graminis* (*Pm*). This gene also confers a leaf tip necrosis phenotype (Ltn) on flag leaves when plants are field‐grown (Dyck, 1991; Hulbert *et al*., 2007; Lagudah *et al*., 2006; Risk *et al*., 2012; Shah *et al*., 2011). Similarly, the *Lr67/Yr46/Sr56/Pm39/Ltn3* gene (hereafter referred to as *Lr67*) shows broad‐spectrum, partial resistance to rust and mildew pathogens (Herrera‐Foessel *et al*., 2014). In contrast, the *Yr36* APR gene provides *P. striiformis* f. sp. *tritici* resistance only (Uauy *et al*., 2005).

The molecular basis of APR is poorly understood when compared with NLR‐mediated resistance. The three APR genes described above have been cloned and encode an ABC‐type transporter (*Lr34*), a hexose transporter (*Lr67*) and a protein kinase fused to a START domain (*Yr36*) (Fu *et al*., 2009; Krattinger *et al*., 2009; Moore *et al*., 2015). The Lr67 protein may act as a dominant negative regulator of hexose transport (Moore *et al*., 2015), while the Yr36 protein increases chloroplast H_2_O_2_ accumulation by phosphorylation of a thylakoid‐associated ascorbate peroxidase (Guo *et al*., 2015). The mode of action of the Lr34 transporter and molecules it transports are unknown. The deletion of a single phenylalanine codon in the D genome‐encoded *lr34*‐susceptible allele converts it to a functional *Lr34* resistance gene (Chauhan *et al*., 2015). This mutation is believed to have occurred after the domestication of wheat (Krattinger *et al*., 2013).

*Lr34* has been used extensively in hexaploid wheat cultivation for many decades and remained durable to all pathogen races over this long period of time (Dyck *et al*., 1966; Kolmer *et al*., 2008). It provides partial resistance that is insufficient to prevent yield losses from rust diseases unless supplemented with additional resistance genes. *Lr34* partial resistance occurs in mature plants (60 days postgermination) and is often associated with Ltn (Dyck, 1991; Singh, 1992a). Ltn, which appears to be accelerated leaf senescence, is particularly apparent in flag leaves (Krattinger *et al*., 2009; Risk *et al*., 2012) and has been used as a phenotypic marker for *Lr34* identification (Shah *et al*., 2011). Ltn, however, is influenced by both the environment and genetic background (Shah *et al*., 2010, 2011; Singh, 1992a). *Lr34* resistance occurs in mature flag leaves after the onset of Ltn (Krattinger *et al*., 2009) with leaf tips showing the greatest resistance (Hulbert *et al*., 2007). Flag leaf tips show up‐regulation of abiotic stress‐responsive genes in the absence of pathogen infection and higher levels of pathogenesis‐related (PR) protein expression upon *P. triticina* infection (Hulbert *et al*., 2007). This apparent abiotic stress response in uninfected, mature *Lr34* tissue is suggested to prime the plant defence response for elevated expression upon rust pathogen challenge (Hulbert *et al*., 2007).

Expression of *Lr34* in transgenic barley seedlings results in a very deleterious phenotype due to the induction of rapid, developmental leaf senescence (Risk *et al*., 2013). Unlike *Lr34* wheat plants, these barley plants show constitutive induction of defence pathways in the absence of pathogen infection (Chauhan *et al*., 2015) and are resistant to pathogens at the seedling and adult plant stage (Risk *et al*., 2013). Barley does not contain orthologous *Lr34* sequences (Krattinger *et al*., 2011), and co‐expression of the *lr34*‐susceptible allele appears to help attenuate negative effects in this species (Chauhan *et al*., 2015).

In contrast to barley, rice encodes an *Lr34* orthologue (Krattinger *et al*., 2011). Expression of the wheat *Lr34* gene in rice was associated with early leaf tip necrosis and deleterious pleiotropic effects in most cases, although not as extreme as observed in barley (Krattinger *et al*., 2016). In some lines, leaf tip necrosis occurred in seedlings at the two‐leaf stage and plants subsequently showed a severe negative impact on axillary shoot formation, plant vigour and spikelet production (Krattinger *et al*., 2016). However, a single line with low seedling expression was recovered that was only marginally compromised. Remarkably, this gene also provided resistance to *Magnaporthe oryzae*, the hemibiotrophic causal agent of rice blast disease. Deletion of the critical phenylalanine codon present in the rice *Lr34* orthologue did not result in disease resistance (Krattinger *et al*., 2016).

Given the remarkable durability of *Lr34*, we have introduced this hexaploid wheat (ABD) D genome‐encoded gene into durum wheat (AB) by transgenesis as a potentially useful source of disease resistance. Amongst these durum transgenics, several lines showed obvious seedling resistance to leaf rust, stripe rust and powdery mildew diseases, a phenotype not associated with the endogenous *Lr34* gene. A strong correlation between seedling resistance and transgene expressions level was observed. Unlike barley and rice, no deleterious accelerated senescence or developmental phenotypes were observed in these seedlings. *Lr34* is therefore potentially of significant benefit for durum wheat germplasm improvement and, with elevated expression, can provide seedling resistance that is not conferred by the endogenous hexaploid wheat gene.

## Results

*Lr34*‐mediated leaf rust resistance occurs in hexaploid wheat seedlings when grown at a constant 10 °C throughout *P. triticina* challenge (Risk *et al*., 2012). However, these seedlings are not resistant when grown at higher temperatures (Risk *et al*., 2012; Rubiales and Niks, 1995; Singh and Gupta, 1992). *Lr34* cold‐induced seedling resistance was exploited to screen transgenic durum cultivar Stewart plants containing a *Lr34* transgene (Figure S1) for *P. triticina* resistance. T1 seedlings from 12 lines were infected with *P. triticina* and after inoculation grown at a constant 10 °C with a 16‐h light/8‐h dark photoperiod. Potentially resistant progeny were observed in 10 T1 families. On susceptible plants, pustules were present after 20 days postinoculation (dpi), which increased in size by 36 dpi. Resistant seedlings were not immune to *P. triticina*, but had an obvious reduction in pustule size (Figure S2). Homozygous *Lr34* lines were identified using PCR and confirmed by DNA blot analysis of DNAs from 25 T2 seedlings using a restriction enzyme/probe combination that also determined transgene copy number (Figure S3). Four independent homozygous T1 lines were produced that contained a single transgene and potentially showed weak (36‐4), moderate (17‐1, 41‐2) and high (39‐2) levels of seedling leaf rust resistance at 10 °C.

**Figure 1:**
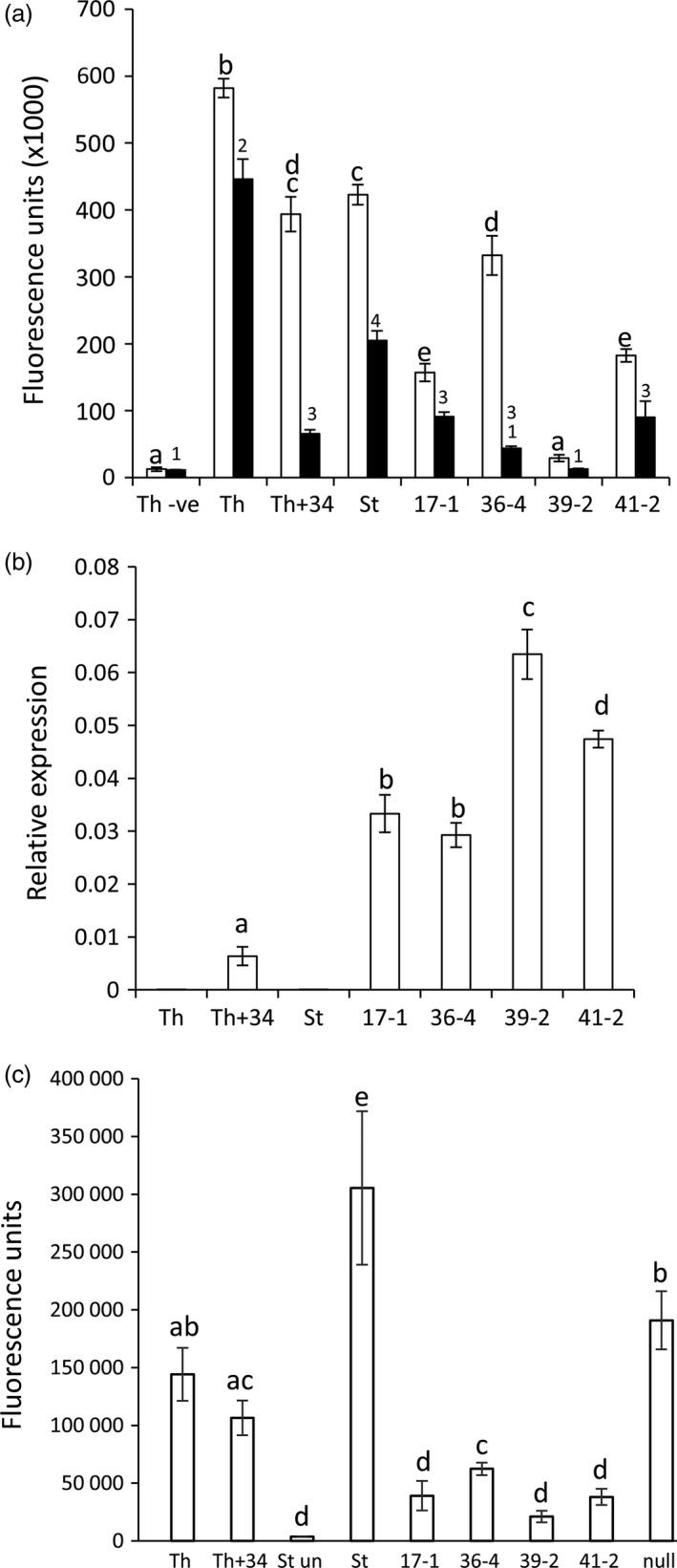
*Lr34* durum resistance corresponds to transgene expression levels. (a) Chitin assay quantification of *P. triticina* growth on wheat seedlings (three‐ to four‐leaf stage) of hexaploid cultivar Thatcher (Th), a near‐isogenic *Lr34* Thatcher line (Th+34), Stewart (St), *Lr34* lines 17‐1, 36‐4, 39‐2, 41‐2 and uninfected Thatcher (Th‐ve). Seedlings were grown at 10 °C (black columns) or 22 °C (white columns) and harvested at 30 and 14 dpi, respectively. Common letters (white columns) or numbers (black columns) indicate data not significantly different (ANOVA,* P* < 0.05), throughout. Each value is the average of four chitin measurements from 10 to 15 pooled seedlings. (b) *Lr34* expression was quantified by Q‐PCR and normalized relative to *GAPDH* in uninfected, three‐ to four‐leaf seedlings of genotypes described in (a). Each data point is derived from three biological replicates, each with three technical replicates. (c) Chitin assay quantification of *P. striiformis* f. sp. *tritici* growth (14 dpi) on 10‐15 seedlings per genotype described in (a) at the three‐ to four‐leaf stage. Using pairwise *t*‐tests (*P* < 0.05), line 39‐2 had significantly less pathogen growth than lines 17‐1 and 41‐2, which each had less growth than line 36‐4. A nontransgenic Stewart line regenerated from tissue culture (null) is included.

Homozygous T2 progeny were re‐screened for seedling leaf rust resistance at 10 °C. For each line, *P. triticina* growth was quantified 30 dpi (Risk *et al*., 2012) by pooling equivalent leaves from 10 to 15 seedlings and the relative amount of fungal chitin present per gram of fresh tissue determined using a chitin assay (Ayliffe *et al*., 2013). Chitin levels were expressed as fluorescence units of bound WGA‐FITC, a fluorophore‐conjugated lectin that specifically binds chitin. A clear reduction in fungal biomass was apparent in homozygous *Lr34* families compared with Stewart control plants (Figure 1a, black columns). Hexaploid wheat cultivar Thatcher carrying the endogenous D genome *Lr34* gene (Th+Lr34) had significantly less rust disease compared with Thatcher control plants, as expected (Figure 1a).

**Figure 2:**
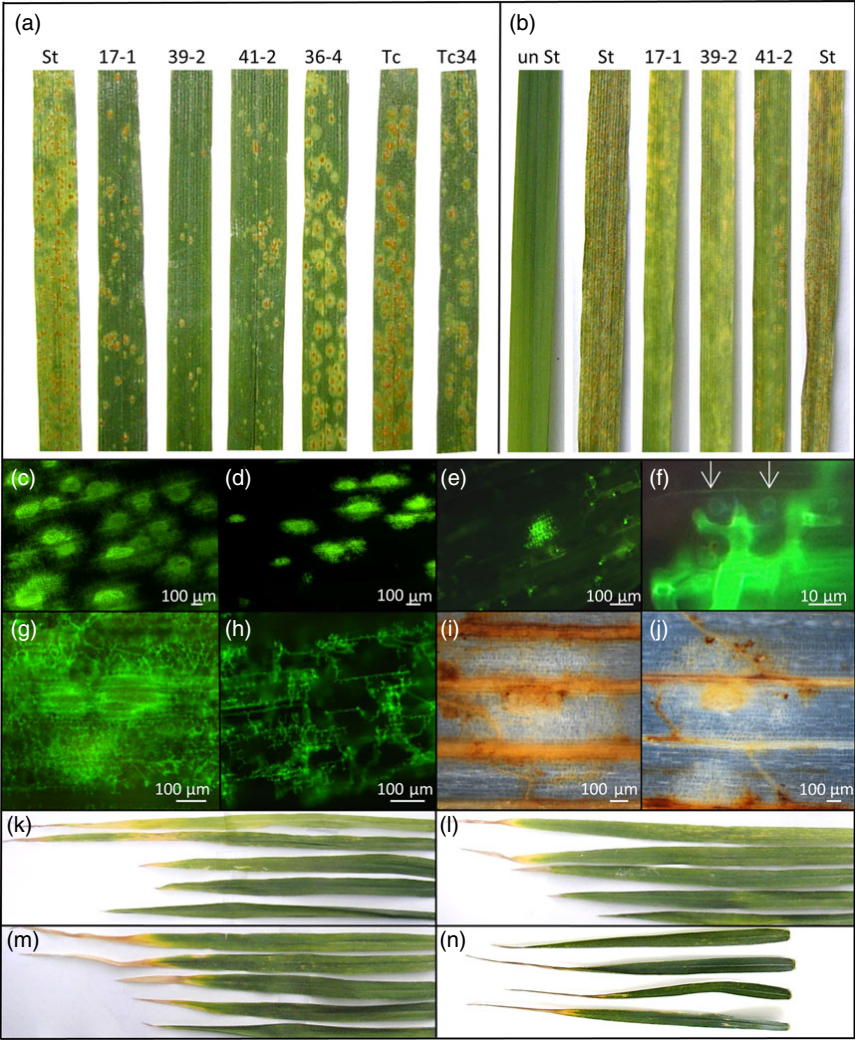
Phenotypic analysis of *Lr34* durum seedlings. (a) *P. triticina* growth (22 °C, 16‐h light) 14 dpi on leaves of seedling of Stewart, *Lr34* lines 17‐1, 39‐2, 41‐2 and 36‐4, Thatcher hexaploid wheat and *Lr34* Thatcher. (b) *P. striiformis* f. sp. *tritici* growth (22 °C, 16‐h light) 14 dpi on *Lr34* seedlings of 17‐1, 39‐2 and 41‐2, uninfected Stewart and infected Stewart. Microscopy of *P. triticina* growth (12 dpi) on Stewart (c) and *Lr34* line 39‐2 (d) stained with WGA‐FITC. (e) *P. triticina* growth (12dpi) on *Lr34* line 39‐2 showing one moderate and numerous small infection sites. (f) *P. triticina* haustoria (arrows) in cells of line 39‐2. (g, h) *P. striiformis* f. sp. *tritici* growth on Stewart (g) and *Lr34* line 39‐2 (h) (WGA‐FITC stained). 3′, 3′‐Diaminobenzidine staining of *P. triticina* infection sites on Stewart (i) and line 39‐2. (j). A brown precipitate shows H_2_O_2_ accumulation in vascular tissue and cells surrounding uredinia (white circle of cells with yellow centre) in each line. Leaves, in descending order of age (i.e. flag leaf at bottom), from glasshouse‐grown Stewart (k), 39‐2 (l) and 41‐2 (m) plants. (n) Flag leaves from field‐grown (top to bottom) Stewart and lines 17‐1, 39‐2 and 41‐2. Tissues in panels c–j were grown at 22 °C, 16‐h light.

These genotypes were then tested for *P. triticina* resistance when plants were grown at 22 °C with a 16‐h light/8‐h dark photoperiod. Under these conditions, Stewart control plants were moderately susceptible (3C on the Stakman scale, where 0 is immune and 4 is highly susceptible (Stakman *et al*., 1962)) to the *P. triticina* isolate used, with obvious macroscopic rust growth and sporulation occurring (Figure 2a). Remarkably, an obvious increase in seedling resistance occurred in T2 seedlings of these durum lines compared with nontransgenic control plants 14 dpi in three replicated experiments (Figures 1a, 2a, 5a, 6a). As expected, Th+34 seedlings had high levels of infection when grown at 22 °C, albeit less than the Tc control (Figures 1a, 2a). The relative levels of rust growth on transgenic seedlings at 22 °C largely mirrored what was seen at 10 °C with line 39‐2 again showing the greatest resistance (Figures 1a, 2a, 5a, 6a), although line 36‐4 was an obvious exception in Figure 1a.

To determine whether a correlation existed between *Lr34* transgene expression levels and the resistance observed in each line, Q‐PCR was undertaken on uninfected leaf tissue of T2 seedlings grown at 22 °C (16‐h light). The PCR primers used amplified transcripts from only Lr34 and not from related homoeologues present on the wheat A genome (no *Lr34* homologue exists on the B genome), or the chromosome 7D *lr34* susceptibility allele of hexaploid wheat (Krattinger *et al*., 2009). A strong correlation occurred between transgene expression levels and disease resistance in these durum lines (Figure 1b). All four lines had significantly greater (4.5‐ to 10‐fold) seedling *Lr34* expression compared with the endogenous Th+34 gene (Figure 1b), presumably explaining the durum seedling resistance observed for this APR gene under glasshouse conditions. Increased *Lr34* expression in these lines is likely due to transgene integration into more transcriptionally favourable regions of the genome.

**Figure 3:**
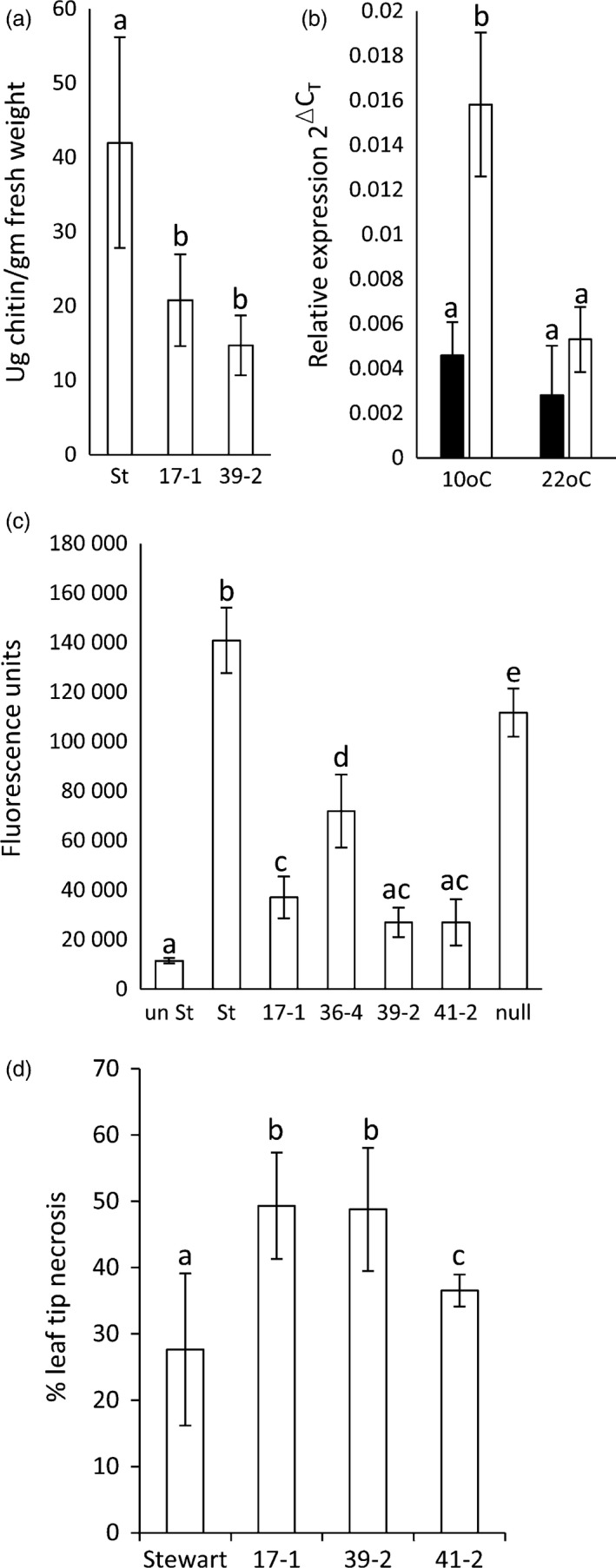
Further characterization of *Lr34* durum lines. (a) Chitin assay quantification of *Blumeria graminis* growth (7 dpi) on leaves of Stewart, 17‐1 and 39‐2. Each data point is from 14 plants and three technical replicates. Fluorescence was converted to ug of chitin/gm of tissue using a standard curve. Data were compared using the Student's *t*‐test, *P* < 0.05. (b) Relative *Lr34* expression in uninfected (black columns) and *P. striiformis* f. sp. *tritici*‐infected (white columns) hexaploid wheat seedlings grown at 10 or 22 °C. Each data point was from pooled leaf tissue (6‐12 seedlings) and triplicate Q‐PCRs. (c) *P. striiformis* chitin assay quantification (26 dpi) of Stewart, 17‐1, 36‐4, 39‐2, 41‐2 and a nontransgenic Stewart line regenerated from tissue culture (null). Seedlings infected at the three‐ to four‐leaf stage were grown at 22 °C (16‐h light) and tissue, when harvested, was undergoing age‐related senescence (Figure S5). Uninfected Stewart seedlings (*un St*) were included. Each data point was from pooled leaf tissue of 12 seedlings and four technical replicates. (d) Ltn on flag leaves of field‐grown *Lr34* durum lines and Stewart. The length of Ltn was divided by total flag leaf length to calculate % Ltn; 10–26 flag leaves were measured per line (see image 2n).

As *Lr34* also provides APR to *P. striiformis* f. sp. *tritici* (wheat stripe rust disease) (Singh, 1992b) and *Blumeria graminis* (wheat powdery mildew disease) (Spielmeyer *et al*., 2005), *Lr34* durum lines were screened for seedling resistance to these pathogens (22 °C, 16‐h light). An obvious reduction in seedling stripe rust growth was observed on transgenic lines both macroscopically and by relative fungal biomass quantification in three replicated experiments (Figures 1c, 2b, S4). The levels of disease resistance again largely correlated with transgene expression levels of each line (Figure 1b). Similarly, significantly less *B. graminis* growth occurred on two *Lr34* durum lines tested with this pathogen using 14 biological replicates per genotype (Figure 3a).

To further investigate the *Lr34* resistance of hexaploid wheat seedlings grown at 10 °C, transcript levels were quantified. Seedlings were grown at 22 °C (16‐h light) and then half transferred to 10 °C growth conditions with the same light regime. After 3 days of acclimation, half of the seedlings in each cabinet were infected with *P. striiformis* f. sp. *tritici* and tissues harvested from infected and uninfected seedlings 3 dpi. A fourfold induction of *Lr34* expression occurred in Tc+34 seedlings grown at 10 °C after *P. triticina* infection (Figure 3b) compared with uninfected seedlings. No equivalent increase in *Lr34* expression occurred in Tc+34‐infected seedlings grown at 22 °C. These data are consistent with similar studies that showed *Lr34* induction in *P. triticina*‐infected wheat seedlings grown at 10 °C, but not in infected seedlings grown under higher temperatures (Risk *et al*., 2012). The ability of *Lr34* to provide seedling resistance in hexaploid wheat at 10 °C therefore appears due to elevated gene expression upon pathogen infection, although additional effects from these growth conditions (e.g. reduced pathogen growth rate and potential cold acclimation response) may contribute to resistance.

Microscopic analyses were undertaken on transgenic durum lines 17‐1, 36‐4, 39‐2 and 41‐2 and control Stewart seedlings after infection with either *P. triticina* or *P. striiformis* f. sp. *tritici*. On all lines, a mixture of infection sites occurred, ranging from small sites to large sporulating uredinia. Control Stewart seedlings showed extensive growth of both pathogens with infection sites usually producing sporulating uredinia (Figure 2c, g), while transgenic durum lines showed less hyphal growth, fewer uredinia and generally smaller infection sites albeit with haustoria (Figure 2d–f, h). Autofluorescent cells were uncommon in all lines infected with either pathogen, suggesting that cell death was not a predominant feature of the resistance response. These data are consistent with previous analysis of adult hexaploid *Lr34* wheat plants after *P. triticina* infection where reduced rust growth occurred without obvious cell death (Risk *et al*., 2012).

As rust growth does still occur on *Lr34* transgenic seedlings, the effect of additional plant growth following *P. striiformis* infection was examined 26 dpi. By this time, infected seedling leaves of all plants were becoming chlorotic and senescent, suggesting that little more growth of this biotrophic pathogen would occur due to leaf ageing (Figure S5). Relative fungal biomass quantification again showed significantly less stripe rust pathogen growth (Figure 3c) on *Lr34* durum seedlings that correlated with transgene expression levels (Figure 1b). These data suggest that rust pathogen growth on these seedlings does not ever reach that observed on nontransgenic control lines.

Expression of *Lr34* in barley causes strong seedling leaf necrosis that appears to be an accelerated senescence response (Risk *et al*., 2013). To assess potential deleterious effects of *Lr34* expression in durum wheat, T3 plants were grown to maturity in the glasshouse. No difference in plant tiller number, tiller height and seed weight yield occurred between control and transgenic durum lines (Figure S6). During plant development, no dramatic differences in leaf senescence rates were apparent (Figure 2k–m). However, under field conditions, transgenic plants did show some increased leaf tip necrosis of flag leaves compared with nontransgenic controls (Figures 2n, 3d). Leaf tip necrosis of mature *Lr34* hexaploid wheat plants is a common phenotype.

**Figure 4:**
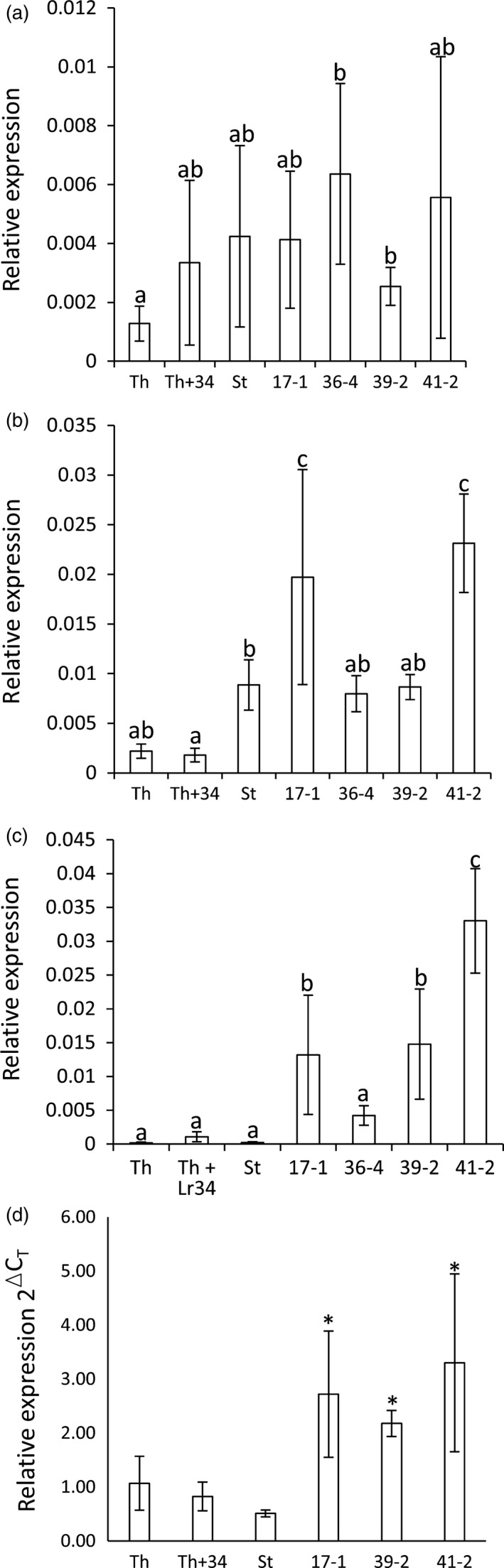
*Lr34* induced gene expression changes in uninfected wheat seedlings. Q‐PCR analyses on RNA from uninfected wheat seedlings (four‐to five‐leaf stage) of hexaploid wheat cultivar Thatcher (Th), a near‐isogenic *Lr34* Thatcher line (Th+Lr34), Stewart and *Lr34* lines 17‐1, 36‐4, 39‐2 and 41‐2. Panels a–d show relative expression of (a) *S40*, (b) *Cp‐MIII*, (c) *Rab15* and (d) *HSP90*, each normalized relative to *GAPDH*. Data were derived from three biological replicates per genotype and three technical replicates per sample. In panel (d), transgenic lines 17‐1, 39‐2 and 41‐2 had significantly higher levels of gene expression (*t*‐test, *P* < 0.05) than the Stewart control, indicated by an asterisk above each column.

To determine whether a weak (i.e. nonvisible) senescence response may be occurring in these transgenic durum seedlings, the first leaves (eldest) were harvested from seedlings, at the fourth leaf stage, and quantification of senescence marker genes was undertaken. Two senescence genes were targeted, *S40* (Krupinska *et al*., 2002) and *CP‐MIII* (*serine carboxypeptidase*) (Parrott *et al*., 2010), with *S40* previously shown to be highly induced in Th+Lr34 flag leaves (Krattinger *et al*., 2009). No significant difference in *S40* expression occurred between Stewart and *Lr34* transgenic lines (Figure 4a). No consistent change in *Cp‐MIII* expression occurred with the most resistant line, 39‐2, and line 36‐4 showing no difference to the Stewart control, while lines 17‐1 and 41‐2 showed a modest increase in expression (two‐ to threefold) (Figure 4b). These data are not consistent with elevated senescence gene expression in *Lr34* durum seedlings and suggest that this seedling resistance is not associated with senescence induction.

An interesting feature of *Lr34* expression in mature bread wheat plants is the up‐regulation of abiotic stress‐responsive genes, predominantly in the tips of flag leaves, in the absence of pathogen infection (Hulbert *et al*., 2007). To determine whether similar gene expression occurred in *Lr34* durum seedlings, expression of abiotic stress‐responsive genes *rab15* (Kosova *et al*., 2014; Tsuda *et al*., 2000) and *HSP90* (Shi *et al*., 2012) was quantified in leaf RNAs of uninfected seedlings grown at 22 °C (16‐h light). A 20‐ to 150‐fold induction of *rab15* occurred in *Lr34* durum seedlings compared with nontransgenic Stewart (Figure 4c), while a more modest four‐ to six‐fold increase in *HSP90* expression occurred (Figure 4d). The expression of *Lr34* in durum seedlings therefore appears to induce an apparent abiotic stress‐related response in the absence of pathogen infection.

**Figure 5:**
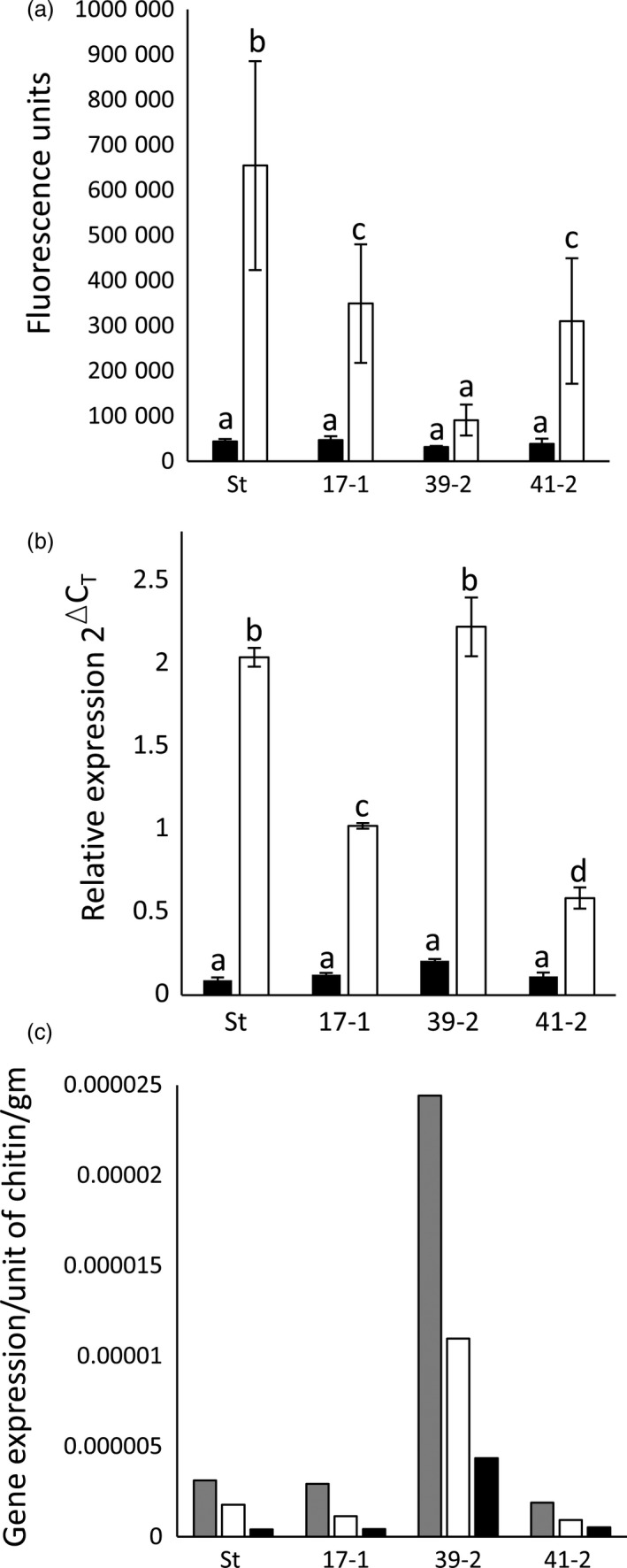
Pathogenesis‐related (*PR*) gene expression in *Lr34* durum seedlings. (a) Chitin assay quantification of uninfected (black columns) and *P. triticina*‐infected (12 dpi) (white columns) Stewart seedlings (St) and lines 17‐1, 39‐2 and 41‐2 (10–15 seedlings per genotype). The same plants were used for RNA extraction in (b). (b) *PR‐1* gene expression in wheat seedlings normalized relative to the *GAPDH*. RNA was extracted from uninfected (black columns) or *P. triticina*‐infected (12 dpi) seedlings (three‐ to four‐leaf stage) described in (a). Three biological replicates were used per genotype and three technical replicates per sample. The same RNAs were quantified for *PR‐2* and *PR‐3* expression (Figure S7). (c) Relative *PR* gene expression values (in B and Figure S7) were divided by chitin biomass values of rust‐infected material shown in A. *PR1*,*PR2* and *PR3* values are shown as grey, white and black columns, respectively, for Stewart (St) and *Lr34* transgenic lines 17‐1, 39‐2 and 41‐2.

Higher *PR* gene expression levels were reported in *P. triticina*‐infected flag leaves of *Lr34* hexaploid plants compared with control lines (Hulbert *et al*., 2007). Transgenic and control durum seedlings were infected with *P. triticina* at the three‐ to four‐leaf stage (22 °C) and tissues harvested 12 dpi. Tissue samples were quantified for both *PR* gene expression and relative fungal biomass. Again, significantly less rust growth occurred in transgenic lines compared with Stewart seedlings (Figure 5a), which correlated with *Lr34* expression levels (Figure 1b). However, no increased expression of *PR1*,* PR2* or *PR3* occurred in *Lr34* durum lines relative to control plants upon rust infection (Figure 5b and Figure S7). These data, however, are complicated by rust infection *per se* strongly inducing *PR* gene expression (Figure 5b and Figure S7: compare uninfected and infected Stewart seedlings). *PR* expression was therefore normalized relative to fungal biomass. Relative to rust growth, strong induction of all three *PR* genes occurred in the most resistant transgenic, line 39‐2, while the remaining more intermediate resistant lines showed no obvious increase in *PR* expression in response to rust infection compared with the control (Figure 5c).

**Figure 6:**
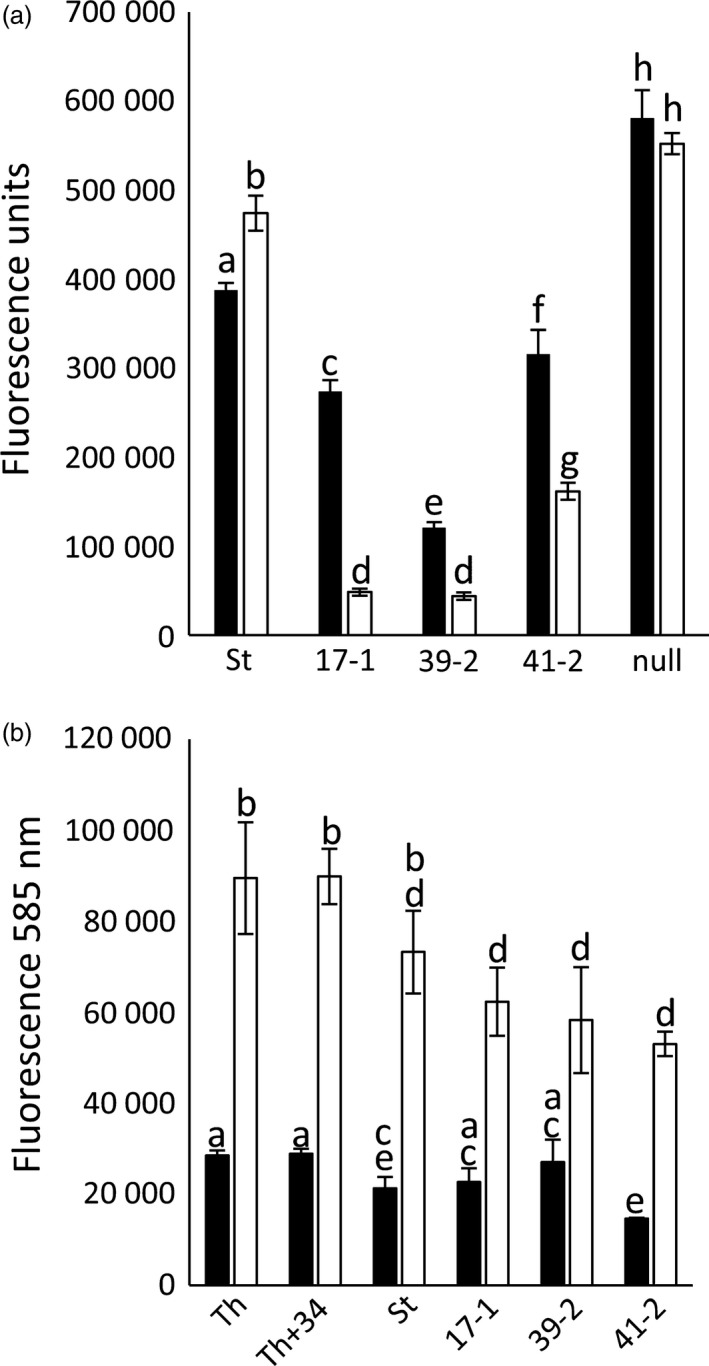
*Lr34* resistance is influenced by photoperiod, but not associated with H_2_O_2_ accumulation. (a) Quantification of *P. triticina* growth (14 dpi) on Stewart (St), *Lr34* lines 17‐1, 39‐2, 41‐2 and a nontransgenic tissue culture regenerant (null) under two photoperiod regimes. Seedlings (four‐leaf stage), grown at 22 °C with a 16‐h light/8‐h dark photoperiod), were infected with *P. triticina* and either returned to these growth conditions (black bars) or grown under constant light (white bars), using the same temperature regime. Each data point is from pooled tissue of approximately six seedlings. (b) Relative H_2_O_2_ levels, determined by Amplex Red assay, in hexaploid wheat cultivar Thatcher (Th), near‐isogenic *Lr34* Thatcher (Th+34), Stewart (St) and *Lr34* durum lines 17‐1, 39‐2 and 41‐2 from infected (white column) or uninfected (black column) plants. Each data point is derived from individual H_2_O_2_ measurements of three biological replicates per genotype.

To examine the influence of photoperiod on *Lr34* resistance, transgenic seedlings were grown at 22 °C (16‐h light) to the three‐ to four‐leaf stage and then inoculated with *P. triticina*. Half of the infected plants were then grown under continuous light, while the remaining seedlings were maintained under a 16‐h light/8‐h dark photoperiod. All *Lr34* lines had significantly less pathogen growth 10 dpi under continuous light when compared with 16‐h light (Figure 6a). Under both conditions, rust resistance levels again correlated with the transgene expression levels (Figure 1b). In contrast, control seedlings showed equivalent or increased rust growth under constant light compared with a 16‐h light/8‐h dark light regime (Figure 6a). Under these highly controlled growth cabinet conditions, increasing photoperiod therefore resulted in increased levels of *Lr34* resistance. It is noteworthy that light has previously been speculated to promote *Lr34* resistance in field‐grown plants (Singh and Gupta, 1992).

Given the light association of *L34* resistance and previous observation that *Yr36* APR results in chloroplast H_2_O_2_ accumulation (Guo *et al*., 2015), H_2_O_2_ accumulation was also examined. Durum seedlings were grown at 22 °C (16‐h light) and H_2_O_2_ content determined in infected and uninfected plants using Amplex Red (Invitrogen). While a clear increase in H_2_O_2_ content occurred upon *P. triticina* infection, no differences in accumulation of this reactive oxygen occurred in resistant and susceptible durum genotypes (Figure 6b). These observations were consistent with 3,3′ diamino benzidine (DAB) staining of H_2_O_2_ in rust‐infected leaf tissue with no obvious difference in H_2_O_2_ accumulation apparent (Figure 2i, J).

## Discussion

Several rust resistance genes have been transferred from durum wheat into hexaploid wheat due to the relative simplicity of crossing these species and selecting fertile hexaploid lines or alternatively producing synthetic hexaploid wheat by crossing tetraploid wheat (AABB) with *Aegilops tauschii* (DD). Germplasm development in tetraploid wheat using interspecies crosses, while achievable (Huguet‐Robert *et al*., 2001; Klindworth *et al*., 2012; Morris *et al*., 2011), is more difficult due to poor vigour and low fertility in tetraploid backgrounds (Ceoloni *et al*., 1996; Klindworth *et al*., 2012). Of particular difficulty is the introduction of D genome genes of hexaploid wheat or *Ae. tauschii* due to the absence of homologous chromosomes in AABB tetraploids and only a few examples have been reported (Ceoloni *et al*., 1996; Han *et al*., 2016; Joppa *et al*., 1998; Liu *et al*., 1996; Luo *et al*., 1996). Given the broad, multipathogen effectiveness and durability of *Lr34*, transfer of this gene to durum wheat by transgenesis is potentially of use in controlling rust and mildew diseases in this crop species.

It was unexpected that this APR gene would also show high levels of seedling resistance in durum wheat which we attribute to transgene expression levels being five to ten times greater than the endogenous gene in hexaploid wheat seedlings, when uninfected seedlings grown at 22 °C were compared. A five‐ to 10‐fold increase in *Lr34* expression also occurs in resistant flag leaves of hexaploid Th+34 plants when compared with seedlings (Risk *et al*., 2012, 2013), consistent with this hypothesis. Importantly, elevated gene expression was not associated with negative pleiotropic effects in durum seedlings and no increased senescence was apparent either macroscopically or by quantification of senescence marker gene transcripts. This seedling resistance further enhances the agronomic potential of *Lr34* in durum wheat cultivation. Seedling resistance is particularly important for protecting wheat from stripe rust disease because it occurs early in the growing season.

Previous introduction of *Lr34* as a transgene into hexaploid wheat cultivar Bobwhite (BW26 AUS) also led to elevated expression levels, with some seedlings showing 10‐fold higher expression; however, seedling resistance was not observed (Risk *et al*., 2012). These lines, however, did show typical *Lr34* APR and minor leaf tip necrosis (Risk *et al*., 2012). Why elevated *Lr34* expression does not provide seedling resistance in hexaploid wheat is unknown. In contrast, *Lr34* transgenics made in a second wheat line, BW26SU, did show seedling resistance to *P. triticina*. This line, however, was not fully rust susceptible and resistance in these lines did not correlate with transgene expression levels. One highly resistant BWSUI line showed only a 2.4‐fold increase in *Lr34* expression relative to Th+34 seedlings. The authors concluded that background resistance present in BWSUI significantly enhanced *Lr34* effects in this line (Risk *et al*., 2012). The *Lr34* gene does show additive effects with minor resistance genes (Singh *et al*., 2011b) and several all‐stage resistance genes (German and Kolmer, 1992).

We cannot exclude the possibility that the *Lr34* resistance in Stewart durum seedlings may be associated with minor gene affects. However, we feel this is improbable. Rust assays showed that Stewart is moderately susceptible to the *P. triticina* (3C rating) and *P. striiformis* f. sp. *tritici* (3, 3+C rating) isolates used in this study (Figure 2a, b), suggesting that minor resistance genes do exist in this background. However, *Lr34* durum lines also had increased powdery mildew disease resistance, meaning minor background genes effective against all three pathogen species would be needed in the Stewart background that showed additive effects with *Lr34*. In addition, resistance levels in these durum lines directly correlated with *Lr34* expression levels consistent with transgene expression being the predominant factor in this seedling resistance.

Elevated *Lr34* expression in durum wheat is well tolerated with only mild leaf tip necrosis in mature plants, although higher expression levels could possibly be deleterious. Hexaploid wheat also tolerates elevated *Lr34* expression, although cold‐grown seedlings showed leaf tip necrosis upon rust infection (which was not observed in durum wheat) and one transgenic line showed chlorotic spotting and reduced seed set (Risk *et al*., 2012). In contrast, similar *Lr34* expression levels in barley result in strong seedling senescence with phenotype severity related to the transgene expression level (Risk *et al*., 2013). Seedling leaf tip necrosis and negative developmental effects were also seen in most *Lr34* rice lines (Krattinger *et al*., 2016).

While *Lr34* resistance usually coincides with accelerated senescence in most plant tissues, including transgenic durum flag leaves, the absence of visible senescence or senescence gene up‐regulation in *Lr34* durum seedlings suggests that senescence is not required for resistance. Consistent with this hypothesis, in rice the amount of leaf tip necrosis in *Lr34* lines did not necessarily correlate with resistance levels (Krattinger *et al*., 2016), implying these two phenotypes are not directly correlated. Ltn is not always apparent in field‐grown, adult *Lr34* plants being dependent on both the environment and genetic background. It is unknown, however, whether plants under these circumstances show senescence up‐regulation without visible necrosis or, alternatively, show no altered senescence response. Hexaploid *Lr34* seedlings grown at 10 °C during rust infection also show resistance, presumably due to elevated *Lr34* expression, without visible accelerated senescence.

This *Lr34* durum seedling resistance, however, is associated with up‐regulation of abiotic stress genes such as *rab15* and *HSP90*, suggesting that this response is required for resistance. Only some of the responses induced by *Lr34* may provide disease resistance and additional effects such as accelerated senescence may be pleiotropic. Common signalling pathways between senescence and abiotic stress responses are well established (Gepstein and Glick, 2013). Some evidence of modest up‐regulated of *PR* expression was observed in rust‐infected durum seedlings although it was confounded by rust infection *per se* causing *PR* induction. Other analyses have shown limited *PR* induction in *Lr34* hexaploid wheat (Risk *et al*., 2012), which differs from the observations of Hulbert *et al*. (2007).

**Figure 7:**
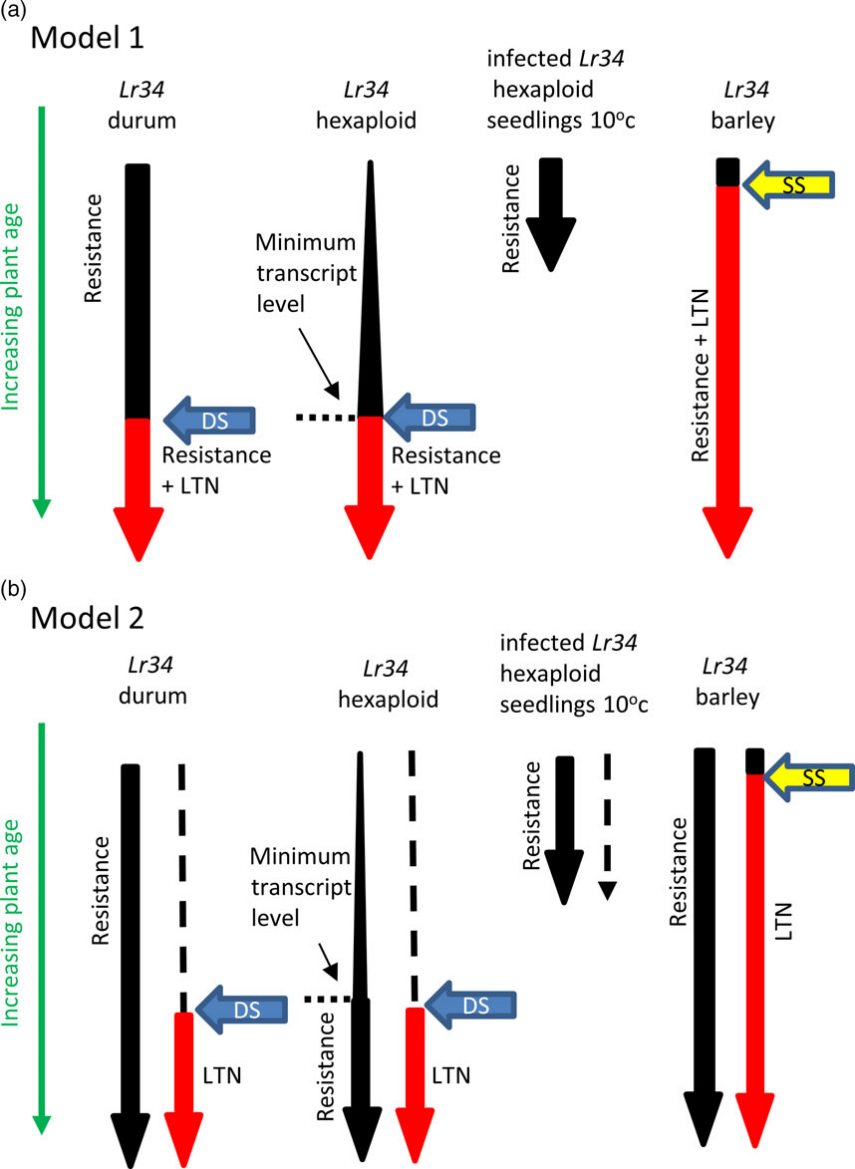
*Lr34* expression and phenotype models. (a) *Lr34* resistance requires a minimum transcriptional threshold (expression levels are depicted by arrow width), which is not reached in hexaploid wheat until later in plant maturity. In contrast, *Lr34* durum seedlings, *Lr34* barley seedlings and cold‐treated, infected hexaploid *Lr34* seedlings are resistant due to higher *Lr34* expression levels. *Lr34* senescence, or leaf tip necrosis (Ltn) (shown in red), is mechanistically the same pathway as resistance but also dependent upon *d*evelopmental *s*ignals (blue arrow labelled DS) that occur later in plant maturation. Hence, cold‐grown, infected *Lr34* hexaploid seedlings and *Lr34* durum seedlings do not show leaf senescence, while adult plants of the same genotypes do. In *Lr34* barley seedlings, signalling during normal developmental *s*enescence of *s*eedling leaves (yellow arrow SS) is sufficient to induce accelerated necrosis due to the heterologous nature of the wheat *Lr34* gene. (b) This model is based upon the same assumptions except that *Lr34* senescence is considered an independent pathway to resistance.

These data raise several possible models for *Lr34* function in durum wheat. In the first scenario, *Lr34* resistance and senescence are mechanistically related, but additional factors, regulated by plant development and the environment, are required for the latter response to occur (Figure 7a). In the case of barley, which does not have an *Lr34* ortholog and may therefore lack appropriate regulatory control of *Lr34*‐mediated processes, these additional factors are inappropriately produced or recognized during initiation of age‐dependent seedling leaf senescence resulting in accelerated leaf necrosis. In hexaploid wheat, a minimum transcriptional threshold needed for *Lr34* resistance is not reached until later in plant development at which time both resistance and senescence occur concomitantly. Consistent with this model, elevated expression of *Lr34* in durum seedlings and in cold‐grown, infected, hexaploid seedlings results in resistance without leaf senescence (Figure 7a). In the second scenario (Figure 7b), the resistance mechanism conferred by *Lr34* is independent of an *Lr34*‐mediated senescence response, which only occurs in tissues of mature plants after reaching a specific developmental age.

*Lr34* durum lines that show robust seedling resistance when grown under field‐like conditions will be a valuable resource to further investigate the mechanistic basis underlying resistance. These transgenic lines demonstrate a clear photoperiod effect on *Lr34* resistance. These observations were of particular interest given the proposed mechanism of the Yr36 START‐kinase protein that phosphorylates a chloroplast thylakoid‐associated ascorbate peroxidise (Guo *et al*., 2015). Elevated H_2_O_2_ levels occur in *Yr36* transgenic plants and accelerated leaf senescence of older leaves (Guo *et al*., 2015). However, no increased H_2_O_2_ accumulation occurred in either uninfected or rust‐infected *Lr34* durum seedlings, suggesting a potentially different mode of action.

In summary, *Lr34* resistance in durum seedlings is not associated with necrosis or accelerated senescence. In contrast, induction of abiotic stress‐response genes occurred in the absence of pathogen infection in these seedlings as previously observed in adult hexaploid wheat plants (Hulbert *et al*., 2007). Photoperiod had a significant effect on *Lr34* phenotypes by as yet undefined mechanisms. Manipulation of APR gene expression can enhance disease resistance without associated negative pleiotropic effects in some instances, which is of potential agronomic benefit.

## Experimental procedures

### Plant and pathogen growth conditions

Wheat plants were grown in growth cabinets at 22 °C, 16‐h light/8‐h dark unless otherwise specified. Wheat seedlings were infected with *Puccinia striiformis* f. sp. *tritici* isolate accession number 821559 (pathotype 104 E137 A‐) and *Puccinia triticina* isolate accession number 020281 (pathotype 104‐1,2,3,(6),(7),11 + Lr37) obtained from the Plant Breeding Institute, NSW, Australia. Plants were inoculated with *P. striiformis* and *P. triticina* urediniospores and incubated in a humid chamber overnight at 10 or 22 °C, respectively. Infected plants were then transferred to growth cabinets (22 °C, 16‐h light/8‐h dark) for growth of rust pathogens. Extended growth times were used for experiments at 10 °C due to significantly slower rust growth (Risk *et al*., 2012). Rust inoculum was propagated on wheat cultivar Morocco and urediniospores collected from infected plants by shaking plants over aluminium foil. For infection with *B. graminis*, durum leaves were harvested from 26‐day‐old seedlings and placed on MS salt media and infected with *B. graminis* isolate ISR208; 7 dpi *B. graminis* growth was measured by chitin assay (Ayliffe *et al*., 2013) using 14 biological replicates per genotype.

### Generation of homozygous *Lr34* transgenic durum wheat plants

Transgenic Stewart durum wheat plants were generated by Agrobacterium‐mediated transformation of cultivar Stewart (Ishida *et al*., 2013; Richardson *et al*., 2014). The *Lr34* transgene (Figure S1A) was cloned into binary plasmid pWBVec8 (Murray *et al*., 2004; Wang *et al*., 1998), which encodes a hygromycin phosphotransferase gene. Transgenic plants were selected using 30–50 μg/mL of hygromycin. To identify Stewart plants containing at least one complete *Lr34* transgene, DNA blot analysis was undertaken on T0 plants as previously described (Ayliffe *et al*., 2000). DNAs were restricted with *NotI* and hybridized with a probe encoding 2 kb of the 3′ terminus of the *Lr34* ORF (Table S1). A predicted 16‐kb fragment with homology was identified in lines containing a complete transgene (Figure S1B). T1 plants were screened for potential *Lr34* transgene homozygosity by PCR analysis of 20 individual T2 seeds using *Lr34*‐specific primers (Table S1, ABCTF4N and Lr34plusR). Transgene copy number in each family was then determined, and homozygosity confirmed, by DNA blot analysis of 25 *DraI*‐restricted T2 plant DNAs hybridized with a probe complementary to a 481‐bp fragment of the *Lr34* 3′ untranslated region (3′UTR) (Table S1, Figure S3). Homozygous families were identified for four independent, single‐copy *Lr34* transgenic events, that is 17‐1, 36‐4, 39‐2 and 41‐2.

### Fungal biomass assays

Seedlings (10–15) at the three‐ to four‐leaf stage from either homozygous *Lr34* transgenic lines or nontransgenic control lines were infected with rust urediniospores. Rust‐infected leaves were harvested 10–14 days postinoculation, and relative chitin biomass per mg fresh weight of harvested tissue was determined by pooling seedling leaves of the same genotype and measuring the binding of wheat germ agglutinin–fluorescein isothiocyanate (WGA‐FITC), as previously described (Ayliffe *et al*., 2013). Relative rust biomass/gm fresh weight of leaf tissue was expressed as fluorescence units of bound WGA‐FITC. Four chitin measurements were undertaken on each seedling leaf tissue pool, and average value was presented with standard deviation.

### Quantitative RT‐PCR

RNA was extracted from frozen, ground wheat leaf tissue using a Spectrum Plant Total RNA Kit (Sigma‐Aldrich) and On‐column DNase I Digestion Kit (Sigma‐Aldrich) for genomic DNA removal. cDNA was synthesized using a reverse transcriptase kit (Phusion RT‐PCR Kit; Finnzymes) and Q‐PCR undertaken using a CFX96 real time system and C100 touch thermocycler (Bio‐Rad). Target gene sequences were normalized relative to the wheat *glyceraldehyde‐3‐phosphate dehydrogenase* gene (*GAPDH*) using the comparative C_T_ method (Schmittgen and Livak, 2008) or alternatively target sequence concentration was determined using a standard curve derived from a target fragment linear dilution series, followed by normalization relative to *GAPDH* (Rinaldo *et al*., 2015). Three replicate reactions were undertaken per RNA sample. Primer sequences used for amplification are shown in Table S1. Melting curves for primer pairs used in Q‐PCR analyses are shown in Figure S8.

### Microscopy and H_2_O_2_ quantification

Fungal‐infected tissue was stained with wheat germ agglutinin–fluorescein isothiocyanate (Sigma‐Aldrich, St. Louis) and visualized under blue light (Ayliffe *et al*., 2011). Histochemical detection of H_2_O_2_ in infected leaf tissue was undertaken by 3,3′‐diaminobenzidine (DAB) staining (Sigma‐Aldrich, St. Louis) (Thordal‐Christensen *et al*., 1997). H_2_O_2_ levels were determined in ground leaf tissues using an Amplex Red H_2_O_2_ assay kit as described by the manufacturer (ThermoFisher).

### Data analysis

All data were analysed by ANOVA using an online calculator (http://statistica.mooo.com/) unless otherwise stated in figure legends. Significantly different data points had a *P* < 0.05 unless indicated otherwise in the text. Standard deviations are indicated on graphs. Graph columns with common annotation were not statistically different (ANOVA, *P* < 0.05), throughout.

## Conflict of interest

The authors declare no conflict of interest.

## Author contributions

AR undertook experiments described in Figures 1a, c, 2, 3c, d, 4, 5, 6a. BG undertook experiments in Figures 1c, 2c. RB and SK produced data in Figure 3a. DS and RP screened rust collections to identify and provide a *P. triticina* isolate virulent on durum cultivar Stewart. EL undertook field trial results in Figure 3d and provided experimental design planning. MA contributed images in Figure 2, undertook experiments in Figures 2l–o and 6b in addition to providing experimental design.

## Supporting information


**Figure S1** Production of *Lr34* transgenics in durum wheat cultivar Stewart.
**Figure S2** *P. triticina* infection phenotypes on durum wheat seedlings grown at 10 °C.
**Figure S3** DNA blot analysis of durum cultivar Stewart transgenic lines containing single‐copy insertions of the hexaploid wheat *Lr34* gene.
**Figure S4** Replicate experimental quantification of stripe rust growth on *Lr34* durum lines.
**Figure S5** Example of tissue harvested from stripe rust‐infected durum seedlings 26 dpi.
**Figure S6** Growth characteristics of *Lr34* transgenic durum wheat lines.
**Figure S7** *PR2* and *PR3* gene expression in *Lr34* durum lines.
**Figure S8** Melt peak curves of Q‐PCR reactions undertaken.

**Table S1** Primers used for Q‐PCR and probe amplification.
