# Supplementary material for: The Lr34 adult plant rust resistance gene provides seedling resistance in durum wheat without senescence
Source: Plant Biotechnol J. 2017 Mar 10;15(7):894–905. doi: 10.1111/pbi.12684 (PMC5466443; doi:10.1111/pbi.12684)
Supplement: Supplementary file 1 — Figure S1 Production of Lr34 transgenics in durum wheat cultivar Stewart. Figure S2 P. triticina infection phenotypes on durum wheat seedlings grown at 10 °C. Figure S3 DNA blot analysis of durum cultivar Stewart transgenic lines containing single‐copy insertions of the hexaploid wheat Lr34 gene. Figure S4 Replicate experimental quantification of stripe rust growth on Lr34 durum lines. Figure S5 Example of tissue harvested from stripe rust‐infected durum seedlings 26 dpi. Figure S6 Growth characteristics of Lr34 transgenic durum wheat lines. Figure S7 PR2 and PR3 gene expression in Lr34 durum lines. Figure S8 Melt peak curves of Q‐PCR reactions undertaken. [file PBI-15-894-s002.pptx]

## Slide 1
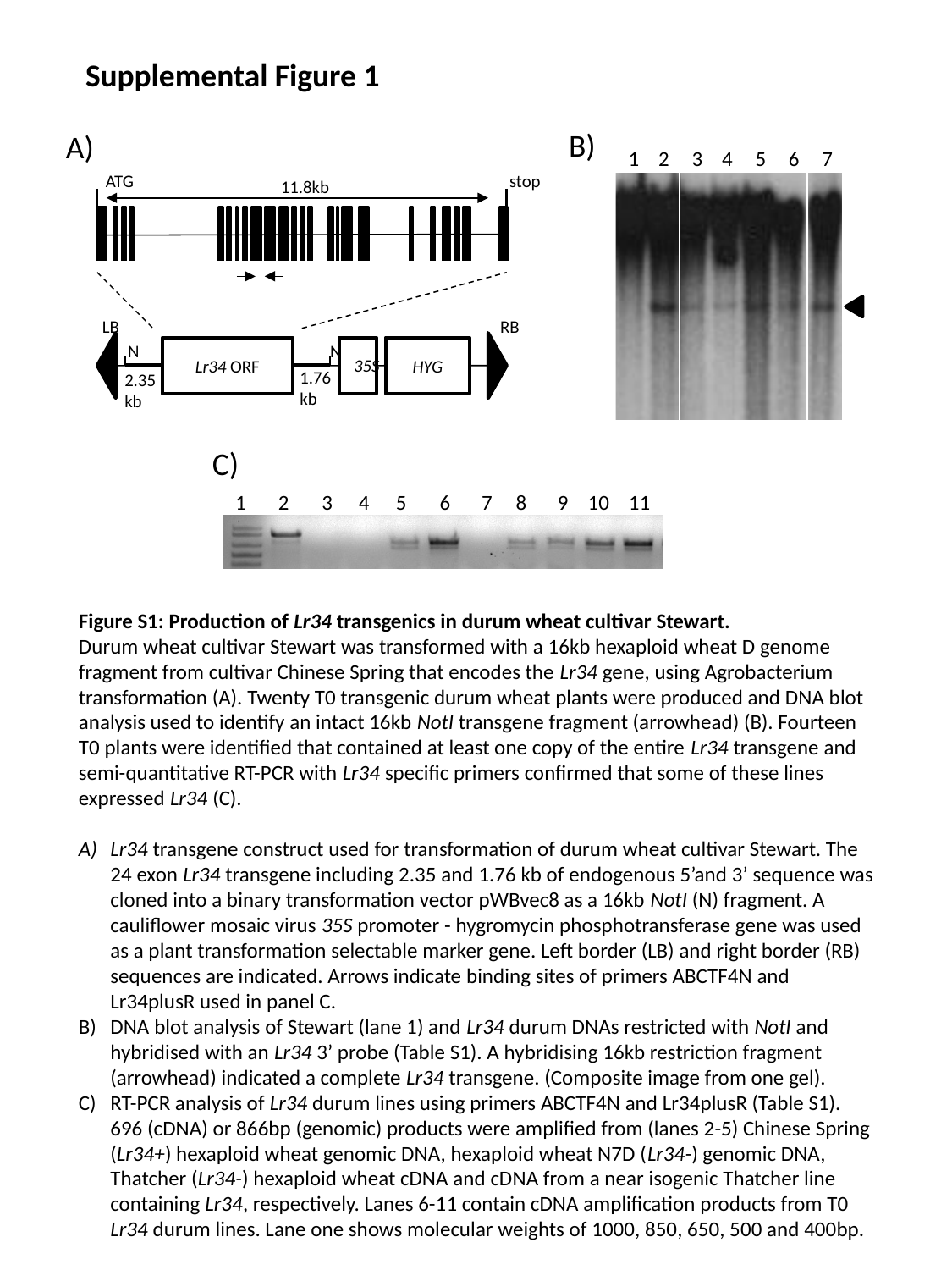

Supplemental Figure 1
B)
1
2
3
4
5
6
7
A)
ATG
stop
11.8kb
LB
RB
N
N
Lr34 ORF
HYG
35S
1.76
kb
2.35
kb
C)
1
2
3
4
5
6
7
8
9
10
11
Figure S1: Production of Lr34 transgenics in durum wheat cultivar Stewart.
Durum wheat cultivar Stewart was transformed with a 16kb hexaploid wheat D genome fragment from cultivar Chinese Spring that encodes the Lr34 gene, using Agrobacterium transformation (A). Twenty T0 transgenic durum wheat plants were produced and DNA blot analysis used to identify an intact 16kb NotI transgene fragment (arrowhead) (B). Fourteen T0 plants were identified that contained at least one copy of the entire Lr34 transgene and semi-quantitative RT-PCR with Lr34 specific primers confirmed that some of these lines expressed Lr34 (C).
Lr34 transgene construct used for transformation of durum wheat cultivar Stewart. The 24 exon Lr34 transgene including 2.35 and 1.76 kb of endogenous 5’and 3’ sequence was cloned into a binary transformation vector pWBvec8 as a 16kb NotI (N) fragment. A cauliflower mosaic virus 35S promoter - hygromycin phosphotransferase gene was used as a plant transformation selectable marker gene. Left border (LB) and right border (RB) sequences are indicated. Arrows indicate binding sites of primers ABCTF4N and Lr34plusR used in panel C.
DNA blot analysis of Stewart (lane 1) and Lr34 durum DNAs restricted with NotI and hybridised with an Lr34 3’ probe (Table S1). A hybridising 16kb restriction fragment (arrowhead) indicated a complete Lr34 transgene. (Composite image from one gel).
RT-PCR analysis of Lr34 durum lines using primers ABCTF4N and Lr34plusR (Table S1). 696 (cDNA) or 866bp (genomic) products were amplified from (lanes 2-5) Chinese Spring (Lr34+) hexaploid wheat genomic DNA, hexaploid wheat N7D (Lr34-) genomic DNA, Thatcher (Lr34-) hexaploid wheat cDNA and cDNA from a near isogenic Thatcher line containing Lr34, respectively. Lanes 6-11 contain cDNA amplification products from T0 Lr34 durum lines. Lane one shows molecular weights of 1000, 850, 650, 500 and 400bp.

## Slide 2
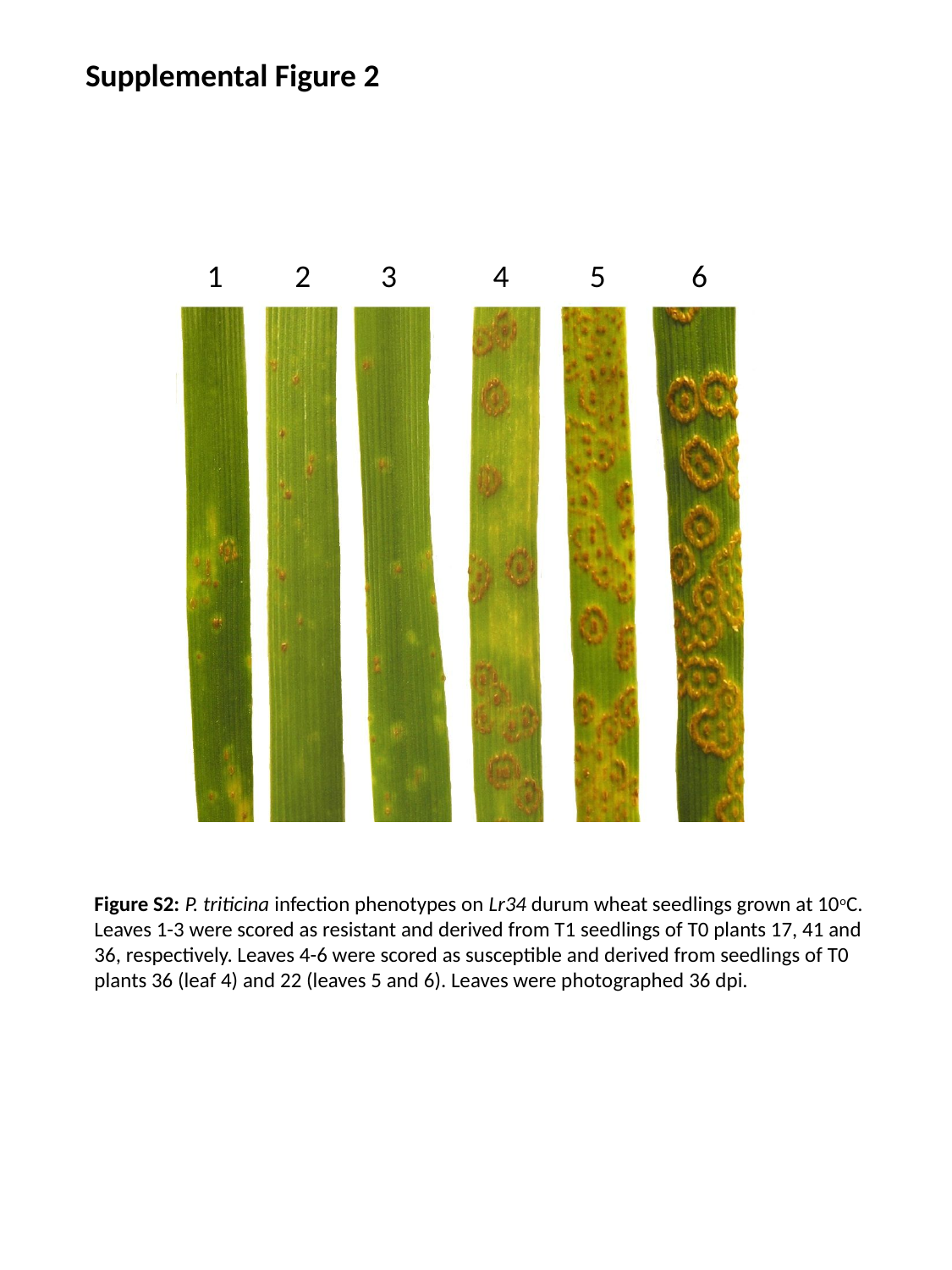

Supplemental Figure 2
1
2
3
4
5
6
Figure S2: P. triticina infection phenotypes on Lr34 durum wheat seedlings grown at 10oC. Leaves 1-3 were scored as resistant and derived from T1 seedlings of T0 plants 17, 41 and 36, respectively. Leaves 4-6 were scored as susceptible and derived from seedlings of T0 plants 36 (leaf 4) and 22 (leaves 5 and 6). Leaves were photographed 36 dpi.

## Slide 3
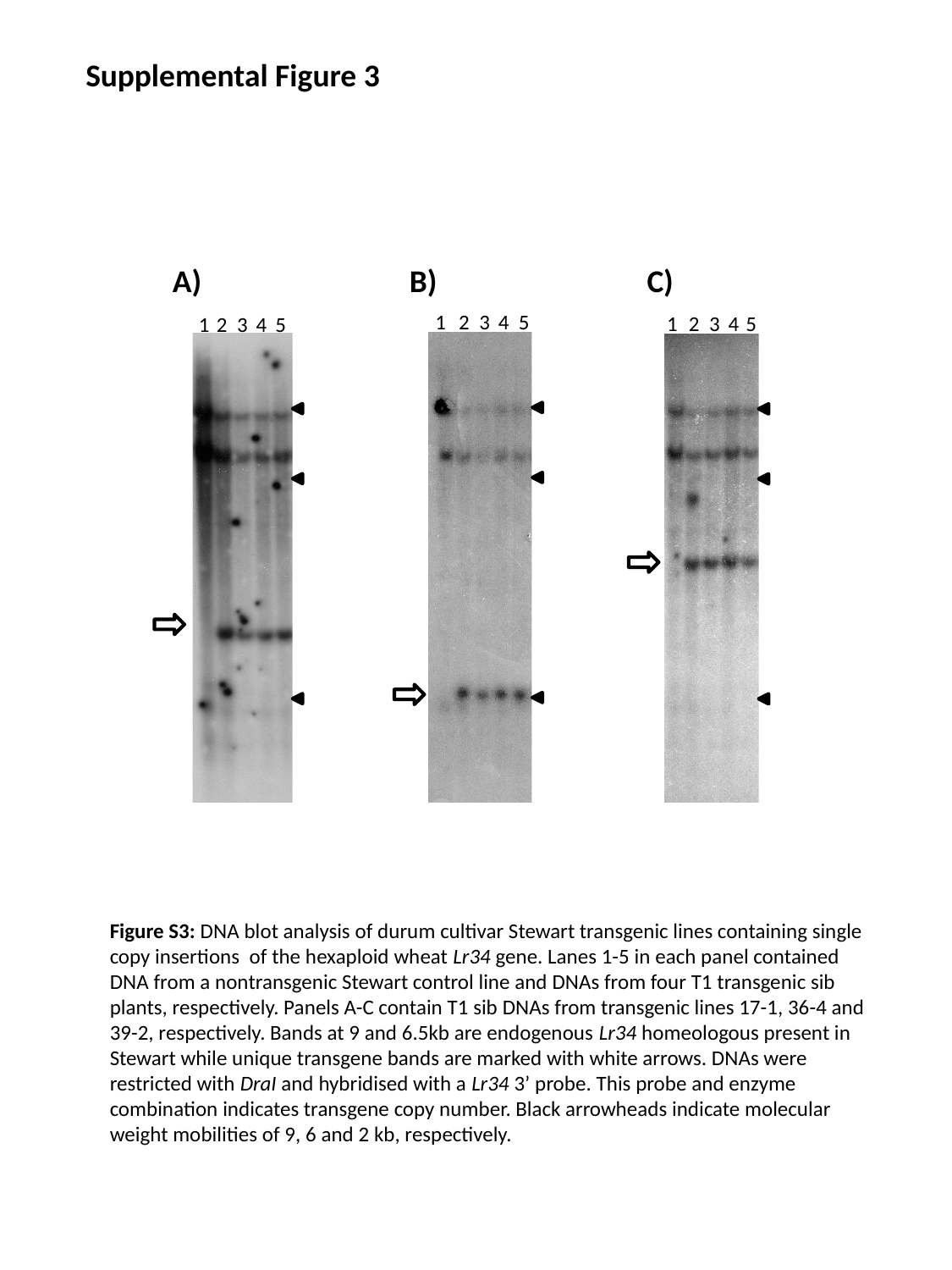

Supplemental Figure 3
A)
B)
C)
1
2
3
4
5
1
2
3
4
5
2
4
1
3
5
Figure S3: DNA blot analysis of durum cultivar Stewart transgenic lines containing single copy insertions of the hexaploid wheat Lr34 gene. Lanes 1-5 in each panel contained DNA from a nontransgenic Stewart control line and DNAs from four T1 transgenic sib plants, respectively. Panels A-C contain T1 sib DNAs from transgenic lines 17-1, 36-4 and 39-2, respectively. Bands at 9 and 6.5kb are endogenous Lr34 homeologous present in Stewart while unique transgene bands are marked with white arrows. DNAs were restricted with DraI and hybridised with a Lr34 3’ probe. This probe and enzyme combination indicates transgene copy number. Black arrowheads indicate molecular weight mobilities of 9, 6 and 2 kb, respectively.

## Slide 4
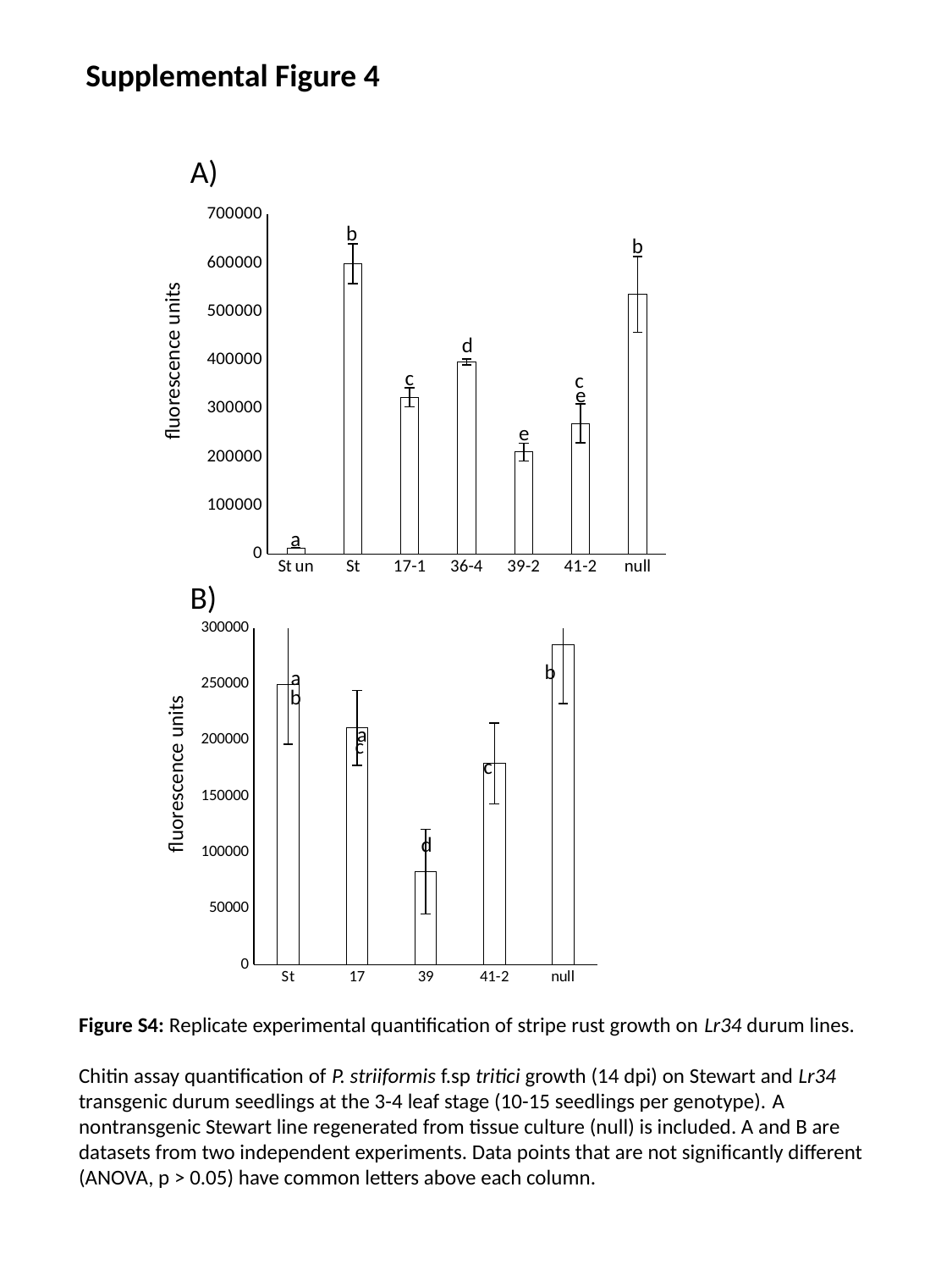

Supplemental Figure 4
A)
### Chart
| Category | |
|---|---|
| St un | 12711.166666666666 |
| St | 598076.6666666666 |
| 17-1 | 322839.1666666667 |
| 36-4 | 396059.3333333333 |
| 39-2 | 210124.5 |
| 41-2 | 269311.1666666667 |
| null | 534955.1666666666 |b
b
c
e
e
d
fluorescence units
c
a
B)
### Chart
| Category | |
|---|---|
| St | 249494.11111111112 |
| 17 | 210945.11111111112 |
| 39 | 82824.33333333333 |
| 41-2 | 179228.33333333334 |
| null | 284608.55555555556 |b
a
b
a
c
c
fluorescence units
d
Figure S4: Replicate experimental quantification of stripe rust growth on Lr34 durum lines.
Chitin assay quantification of P. striiformis f.sp tritici growth (14 dpi) on Stewart and Lr34 transgenic durum seedlings at the 3-4 leaf stage (10-15 seedlings per genotype). A nontransgenic Stewart line regenerated from tissue culture (null) is included. A and B are datasets from two independent experiments. Data points that are not significantly different (ANOVA, p > 0.05) have common letters above each column.

## Slide 5
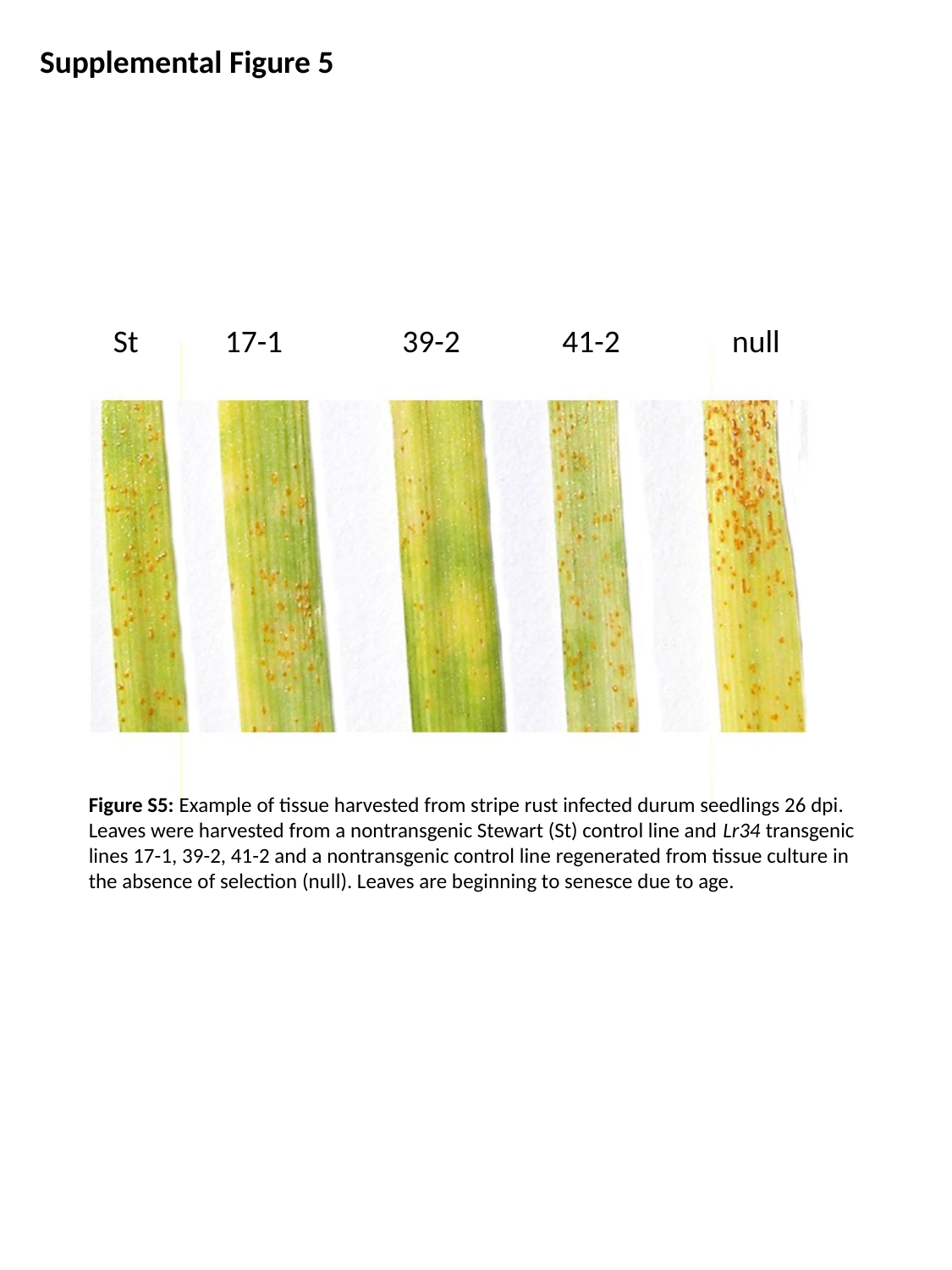

Supplemental Figure 5
St
17-1
39-2
41-2
null
Figure S5: Example of tissue harvested from stripe rust infected durum seedlings 26 dpi. Leaves were harvested from a nontransgenic Stewart (St) control line and Lr34 transgenic lines 17-1, 39-2, 41-2 and a nontransgenic control line regenerated from tissue culture in the absence of selection (null). Leaves are beginning to senesce due to age.

## Slide 6
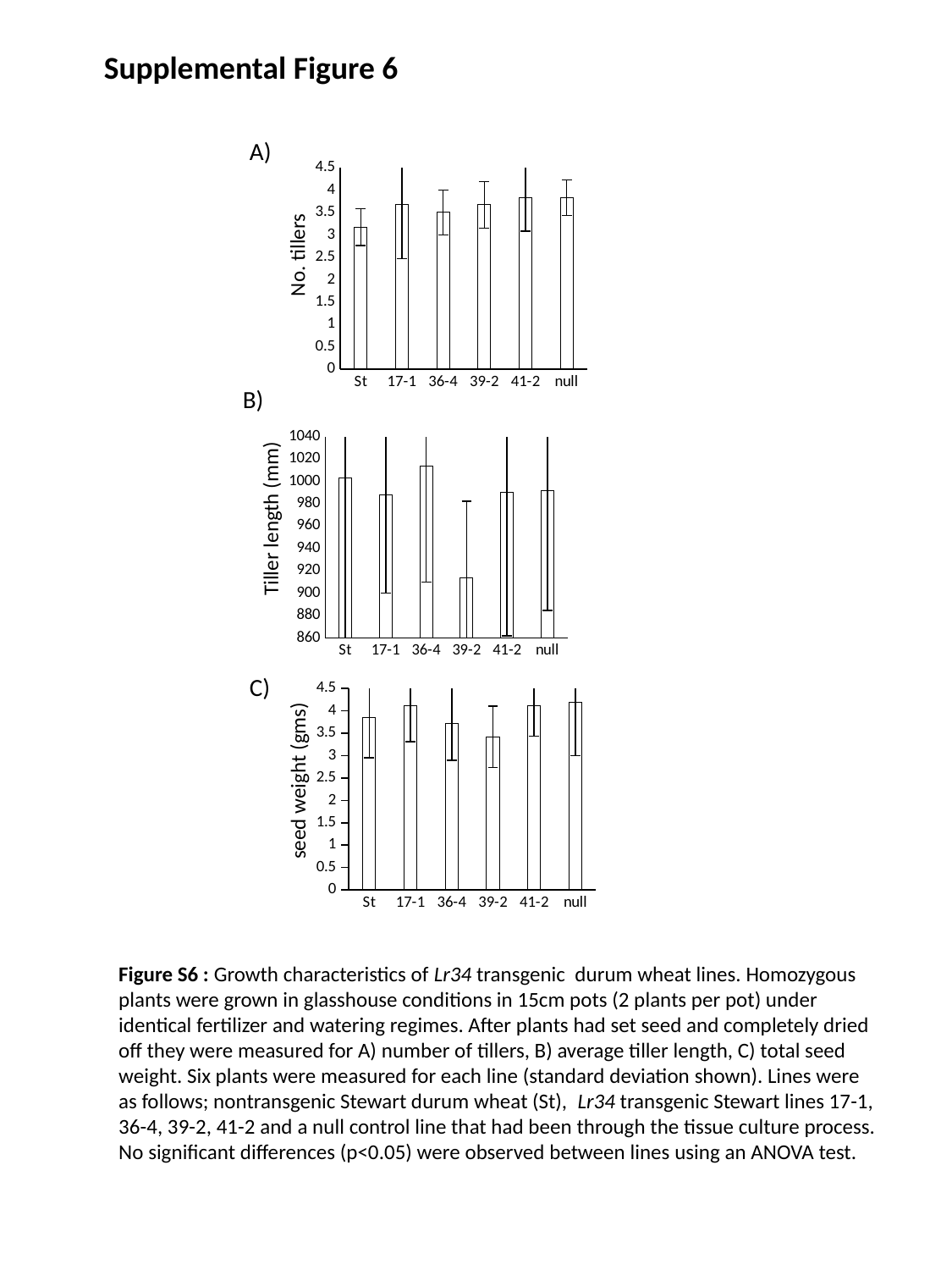

Supplemental Figure 6
A)
### Chart
| Category | |
|---|---|
| St | 3.17 |
| 17-1 | 3.67 |
| 36-4 | 3.5 |
| 39-2 | 3.67 |
| 41-2 | 3.83 |
| null | 3.83 |No. tillers
B)
### Chart
| Category | |
|---|---|
| St | 1002.6 |
| 17-1 | 988.0 |
| 36-4 | 1013.7 |
| 39-2 | 913.9 |
| 41-2 | 990.0 |
| null | 991.52 |Tiller length (mm)
C)
### Chart
| Category | |
|---|---|
| St | 3.85 |
| 17-1 | 4.11 |
| 36-4 | 3.72 |
| 39-2 | 3.42 |
| 41-2 | 4.12 |
| null | 4.19 |seed weight (gms)
Figure S6 : Growth characteristics of Lr34 transgenic durum wheat lines. Homozygous plants were grown in glasshouse conditions in 15cm pots (2 plants per pot) under identical fertilizer and watering regimes. After plants had set seed and completely dried off they were measured for A) number of tillers, B) average tiller length, C) total seed weight. Six plants were measured for each line (standard deviation shown). Lines were as follows; nontransgenic Stewart durum wheat (St), Lr34 transgenic Stewart lines 17-1, 36-4, 39-2, 41-2 and a null control line that had been through the tissue culture process. No significant differences (p<0.05) were observed between lines using an ANOVA test.

## Slide 7
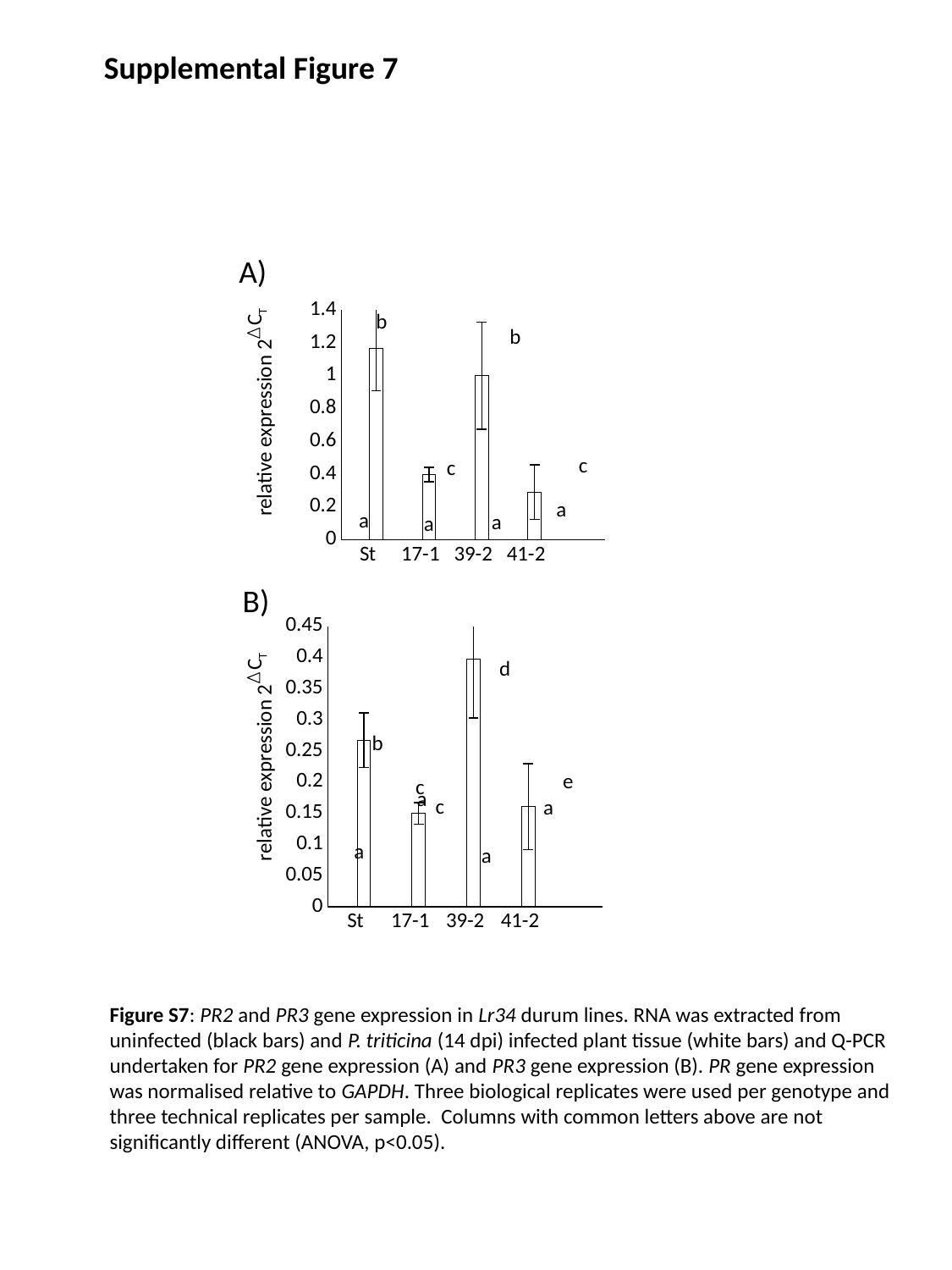

Supplemental Figure 7
A)
### Chart
| Category | | |
|---|---|---|
| St | 0.05590732915423205 | 1.1650294858007255 |
| 17-1 | 0.03338191315147385 | 0.3967677324996177 |
| 39-2 | 0.039170625840637664 | 0.9993479916534427 |
| 41-2 | 0.052499599592640465 | 0.2888037489081475 |B)
### Chart
| Category | | |
|---|---|---|
| St | 0.048617431948077106 | 0.2669911545941294 |
| 17-1 | 0.11342003898035595 | 0.1496346046510647 |
| 39-2 | 0.046921589339097125 | 0.3970414895105166 |
| 41-2 | 0.06409234623945415 | 0.1602909100618424 |b
b
CT
relative expression 2
c
c
a
a
a
a
CT
relative expression 2
c
a
c
a
a
a
d
b
e
Figure S7: PR2 and PR3 gene expression in Lr34 durum lines. RNA was extracted from uninfected (black bars) and P. triticina (14 dpi) infected plant tissue (white bars) and Q-PCR undertaken for PR2 gene expression (A) and PR3 gene expression (B). PR gene expression was normalised relative to GAPDH. Three biological replicates were used per genotype and three technical replicates per sample. Columns with common letters above are not significantly different (ANOVA, p<0.05).

## Slide 8
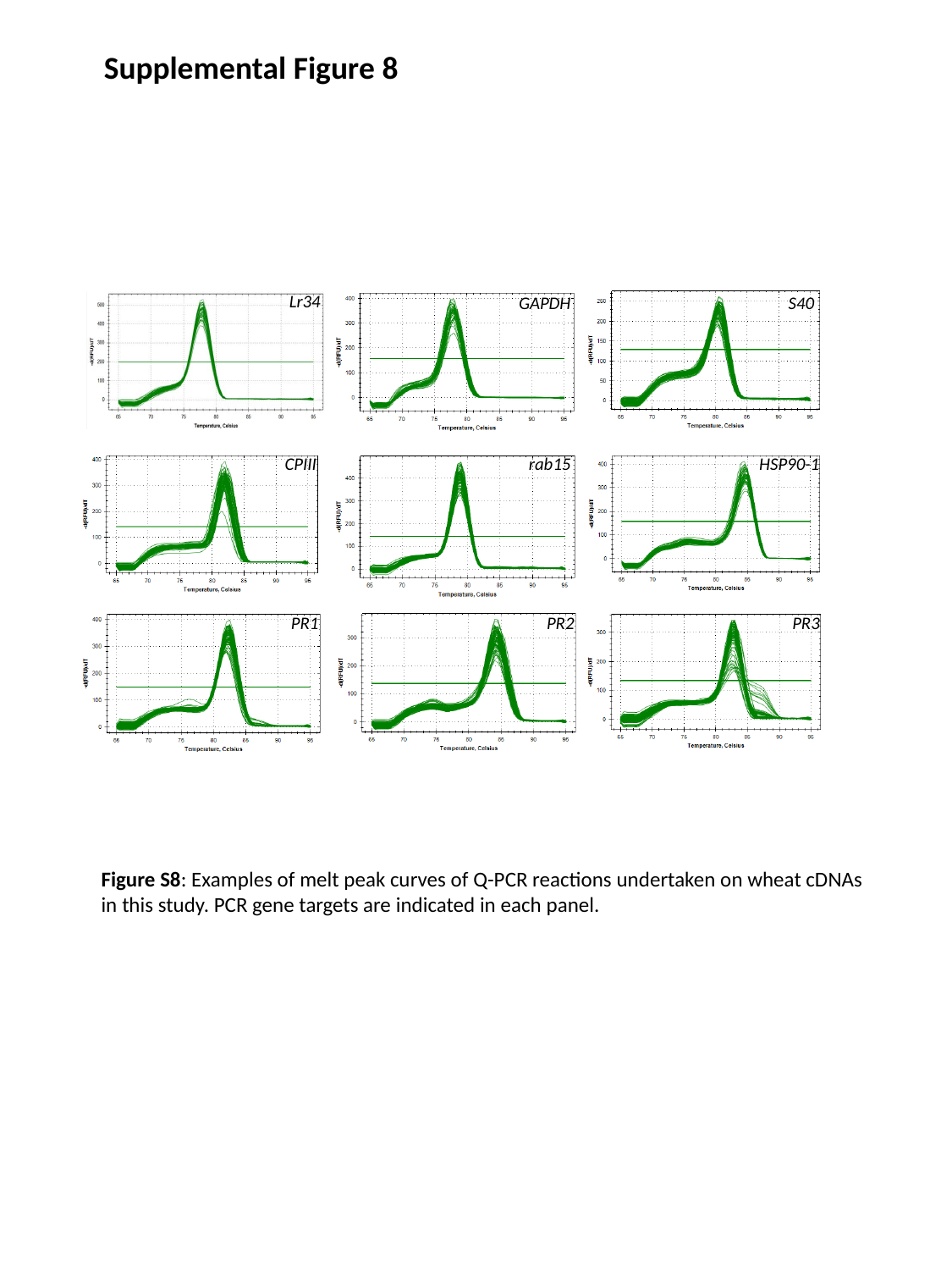

Supplemental Figure 8
Lr34
GAPDH
S40
CPIII
rab15
HSP90-1
PR1
PR2
PR3
Figure S8: Examples of melt peak curves of Q-PCR reactions undertaken on wheat cDNAs in this study. PCR gene targets are indicated in each panel.
