# Supplementary material for: The Lr34 adult plant rust resistance gene provides seedling resistance in durum wheat without senescence
Source: Plant Biotechnol J. 2017 Mar 10;15(7):894–905. doi: 10.1111/pbi.12684 (PMC5466443; doi:10.1111/pbi.12684)
Supplement: Supplementary file 2 — Table S1 Primers used for Q‐PCR and probe amplification. [file PBI-15-894-s001.docx]

**Supplemental Table S1: Primers used for Q-PCR and probe amplification**

| **Gene target** | **Product** | **GenBank ID** | **Primers** |
| --- | --- | --- | --- |
| *CP-III* | serine carboxypeptidase III gene | AK331101 | P1: TCGACGACCACGTTTTACGC  P2:ACAGCAGACACTCCTTGAGA |
| *S40* | senescence associated nuclear protein | AM939937 | P1: AAAGGGAGGACGCTCAAAGG  P2: TCATTTCTCGATGAATCCGGT |
| *Rab15B* | small GTP binding protein | X62476 | P1: CCACCATACAACGTGAGCCT  P2: CGCGAGGACCATACCGTAAA |
| *GAPDH* | glyceraladehyde-3-phosphate dehydrogenase | AF251217 | P1: TTAGACTTGCGAAGCCAGCA  P2: AAATGCCCTTGAGGTTTCCC |
| *Lr34res* | ABC transporter – resistant allele | FJ436983 | P1: GGGAGCATTATTTTTTTCCATCATG  P2: ACTGGCAGAAGAACCTTGAAACA |
| *PR1* | Pathogenesis-related protein 1 | AF384143 | P1: CTGGAGCACGAAGCTGCAG  P2: CGAGTGCTGGAGCTTGCAG |
| *PR2* | Beta-1,3-endoglucanase | AF515785 | P1: CGTAATGCGGCATCCAAGT  P2:GCATGCTTGGTTGCACTCTTC |
| *PR3* | Chitinase 1 | AK367847 | P1:TCTGACGCCCATCACAGTGTAT  P2:CCACGATGGCATATGTACGGTAT |
| *Lr34* 3’ probe | 2 kb amplicon encoding the 3’ terminal 1954bp of the of *Lr34* ORF plus 60bp of 3’UTR | FJ436983.1 | P1:CAGAACACCTACAGAAGAATATC P2:GGCAAGTAGCTATATCTGTAAC |
| *Lr34* | Nucleotides 958-1653 of *Lr34* ORF sequence | FJ436983.1 | Primer ABCTF4N: ATGATTGTGGGCCCCGCAAGT  Primer Lr34plusR:  GCCATTTAACATAATCATGATGGA |
| *Lr34* 3’ UTR probe | 481bp fragment encoding the *Lr34* 3’ UTR sequence | FJ436983.1 | P1: GGTGAACATGATCCGCAAC  P2: GCACCTTAGCCATGAATC |
